# Corticosterone Concentration Varies With Metabolic Rate in Vertebrates

**DOI:** 10.1002/ece3.74243

**Published:** 2026-08-27

**Authors:** Alyson R. Wisnionski, Catherine G. Haase, Isabella Dy, Kyle Hudson, James F. Gillooly

**Affiliations:** ^1^ Department of Biology University of Florida Gainesville Florida USA; ^2^ Department of Biology Austin Peay State University Clarksville Tennessee USA

**Keywords:** allometric scaling, glucocorticoids, metabolic theory, physiological stress, stress hormones

## Abstract

Characterizing the adaptive or maladaptive effects of physiological stress based on glucocorticoid hormone levels remains a challenge in vertebrates. It remains unclear to what extent these measures capture the multi‐dimensional nature of physiological stress, how to compare measures across species, and how to quantify the cumulative effects of stress on health or longevity. To address these issues, we aim to characterize the relationship of glucocorticoid hormone levels to body mass, metabolic rate, and lifespan across species of vertebrates. Specifically, we focus on investigating these relationships for the glucocorticoid hormone corticosterone using data from 160 species of reptiles, birds, and mammals. Results from each vertebrate class show that plasma corticosterone increases with mass‐specific metabolic rate and declines with increasing body mass like mass‐specific metabolic rate. Moreover, across classes, a strong, positive relationship was observed between elevated and baseline corticosterone values. However, no relationship was observed between corticosterone levels and lifespan after removing the effects of metabolic rate. These results suggest that measures of corticosterone across species largely reflect differences in rates of species' energy use. Understanding to what extent corticosterone is linked to individual energetics represents a step forward in developing a conceptual and quantitative framework for linking stress, energetics, and life‐history traits across species.

## Graphical abstract

Using data from 160 species of reptiles, birds, and mammals, we aim to characterize the relationship of glucocorticoid hormone levels to body mass, metabolic rate, and lifespan across species in an effort to develop a comparative framework and assess cumulative effects of physiological stress. Across vertebrate classes, corticosterone is positively correlated with mass‐specific metabolic rate and declines with increasing body mass; however, corticosterone shows no independent relationship with lifespan after accounting for the effects of metabolic rate. These findings indicate that measures of physiological stress across species largely mirror differences in energy use, advancing a quantitative framework for linking stress physiology, energetics, and life‐history traits.

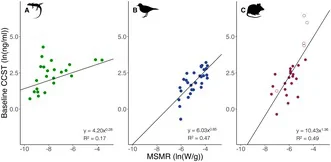

## 1 Introduction

Measuring stress in vertebrates remains a challenge given its multi‐dimensional nature. This impedes our ability to assess both the short‐term benefits and long‐term costs associated with reoccurring stress (Romero et al. 2015). Broadly, physiological stress is recognized as a complex, layered response to a perceived threat or perturbation that leads to short‐term changes in a wide array of physiological and cognitive traits (Romero 2004; McEwen 2000; Levine 2005). This temporary departure from homeostasis may bolster the ability of an individual to respond (Koolhaas et al. 2011). The sympathetic nervous system in coordination with the hypothalamic pituitary adrenal (HPA) axis upregulates a variety of systems to initiate a response to perceived threats, including the cardiovascular, nervous, and musculoskeletal systems (Hermans et al. 2014; de Kloet et al. 2005; McEwen and Wingfield 2003). The response serves to release stored energy, improve performance, and sharpen mental acuity to promote resilience and adaptability (Hermans et al. 2014; de Kloet et al. 2005; McEwen and Wingfield 2003). However, over the long term, physiological stress is generally considered maladaptive. Reoccurring stress episodes may wear down the body's ability to adequately respond to stressors and restore homeostasis (de Kloet et al. 2005; Sapolsky et al. 1990; Romero et al. 2009). More broadly, the cumulative effects of stress may negatively impact survival, growth, or reproduction (McEwen and Wingfield 2003; Sapolsky et al. 1990, 2000). This assumes that species have not evolved ways to mitigate the negative effects of chronic stress through DNA repair mechanisms, reductions in receptor density, or other means (Sapolsky 2021; Bhaskaran et al. 2026).

Physiological stress among vertebrates is most commonly measured based on the concentration of glucocorticoid (GC) hormones, namely cortisol and corticosterone (Romero 2004; Wingfield et al. 1997). While there is growing recognition that GC hormones do not fully capture the multi‐dimensional nature of physiological stress (Romero 2004; Romero and Beattie 2021; Dantzer et al. 2014), they are nevertheless known as “stress hormones” because they are commonly used to quantify species' responses to threat or disturbance (Wingfield et al. 1997; MacDougall‐Shackleton et al. 2019). Typically, in reptiles, birds, and amphibians, corticosterone is the primary GC hormone, whereas in fishes and mammals, cortisol is the primary GC hormone with corticosterone appearing in lower concentrations or not at all (Romero and Butler 2007; Cockrem 2013). However, rodents represent a notable exception among mammals since they primarily rely on corticosterone and produce little to no cortisol (Romero and Butler 2007; Cockrem 2013). These hormones, which are commonly collected from blood, tissue, or fecal samples, are typically measured before and after an individual is exposed to a stressor (e.g., capture/handling, extreme temperatures, threat of predation or injury) to assess the species' response (Guillemin et al. 1958; Koren et al. 2002; Harper and Austad 2000). Predicting the magnitude of this response across species remains a challenge, though previous work in mammals has shown that elevated plasma cortisol levels are highly correlated with baseline levels (Haase et al. 2016).

Two major issues exist with the use of GC hormones in measuring physiological stress. First, there is currently no quantitative framework for comparing stress levels across species (Romero et al. 2015; Moberg 1987). We have few a priori expectations regarding how different species should respond to the same or similar stressor. In part, this is simply because most studies of physiological stress have focused on individuals or populations of the same species. This hinders our ability to broadly consider if and how stress impacts the life histories or fitness of species across communities (Moberg 1987; Palme 2019). Second, it is unclear how to assess the cumulative effects of stress. The use of GC hormone concentrations as a measure of stress presents challenges in considering the effects through time (Romero et al. 2015).

Previous studies investigating the link between GC hormone levels and metabolic rate may be helpful in considering how to overcome the challenges in measuring physiological stress. They have shown that stress hormone concentrations may vary with rates of metabolic energy expenditure (Jimeno et al. 2018; Jimeno and Verhulst 2023). This observation is consistent with the well‐established role of glucocorticoids in blood glucose regulation (Kuo et al. 2015; de Guia et al. 2014). For example, Haase et al. (2016) found that plasma cortisol levels vary in proportion to resting mass‐specific metabolic rate across diverse mammals, and thus vary predictably with body mass. Francis et al. (2018) also found support for a relationship between plasma GC concentrations and body mass across a broad range of vertebrates, although results were mixed within classes. Further understanding the extent to which GC levels and metabolic rate covary could provide a point of departure for establishing a priori expectations in cross‐species comparisons since metabolic rate varies predictably with body size and temperature (Kleiber 1947; Gillooly et al. 2001). This is particularly true in the case of corticosterone since most previous research on this topic has focused on cortisol or general measures of glucocorticoids.

Moreover, understanding the relationship between GC levels (including corticosterone) and metabolic rate may allow one to better understand how and why stress may accumulate through time to impact life history processes, particularly lifespan. Chronic stress is thought to reduce lifespan (Sapolsky et al. 1987; Razzoli et al. 2018). Prolonged and repetitive exposure to stressors that elevate GC levels over the long term is thought to damage cells and accelerate aging by increasing free radical production (Aschbacher et al. 2013; Yegorov et al. 2020; Pickering et al. 2013). Yet, the mechanism linking GC levels and free radical production remains unclear. One possibility is that the relationship between physiological stress and metabolic rate may explain the negative impacts of chronic stress on lifespan since free radical production is a natural byproduct of metabolism (Beckman and Ames 1998; Harman 1956). In other words, the mechanism by which chronic stress affects lifespan may be similar to that of the Free Radical Theory of Aging (Harman 1992; Ames et al. 1993), and consistent with studies showing lifespan is inversely related to mass‐specific metabolic rate (Pearl 1928; McCoy and Gillooly 2008). Examining the relationship between lifespan and GC levels across species, after removing any effects of metabolic rate (or the metabolic component of stress), may help clarify this issue.

Here we build on previous research (Haase et al. 2016; Francis et al. 2018) by focusing exclusively on how corticosterone relates to metabolic rate, body mass, and lifespan across two vertebrate classes that rely primarily on corticosterone (reptiles, birds) and one that relies primarily on cortisol (mammals). Previous studies have focused on how cortisol or total GC concentrations relate to metabolic rate and body mass in vertebrates, and they have not yet considered the relationship of either hormone with lifespan. We begin by first investigating whether plasma corticosterone concentrations vary predictably with metabolic rate, and thus with body mass, across vertebrates. Specifically, we test if corticosterone, CCST (ng/mL), increases proportionally with mass‐specific metabolic rate, MSMR (W/g), and thus declines proportionally with increasing body mass, *M* (g), as predicted by Haase et al. (2016) for cortisol such that:

(1) $$\text{CCST}\propto \text{MSMR}\propto M^{-\frac{1}{4}}$$

Equation (1) predicts a natural log‐transformed plot of corticosterone versus metabolic rate should yield a straight line with a slope of 1, and that a natural log‐transformed plot of corticosterone versus body mass should yield a straight line with a slope of −0.25 (Haase et al. 2016; Gillooly et al. 2002; Brown et al. 2004). Next, we evaluate if plasma corticosterone concentration varies inversely with lifespan after removing any effects of metabolic rate. Finally, we further extend previous research (Haase et al. 2016; Francis et al. 2018) by considering the relationship between baseline and elevated corticosterone levels across these three vertebrate classes (reptiles, birds, and mammals) and baseline corticosterone and cortisol levels in mammals. To date, a relationship between baseline and elevated GC levels has only been shown for cortisol in mammals (Haase et al. 2016).

## 2 Methods

### 2.1 Data Collection

Data were compiled over approximately a 1‐year period beginning July 1, 2024 using Google Scholar. Initially, data were collected haphazardly using keywords such as “stress” or “stress response”, “glucocorticoids”, “corticosterone”, and “birds”, “mammals”, or “reptiles.” In February of 2025, we then performed a second, more systematic search to find papers that we may have missed using the keywords “plasma corticosterone concentration” and “stress response” joined by Boolean operator “and” to yield approximately 150 papers that might contain new sources of data. All sources were then combined and filtered by the following four criteria in this order: First, sources were restricted to studies that used radioimmunoassay (RIA), enzyme immunoassay (EIA), enzyme‐linked immunosorbent assay (ELISA), or double isotope dilution assay (DIDA) to limit variation due to assay type. Second, when multiple sources were available for the same species, papers using EIA were chosen. Third, if there were multiple studies using EIA, the study measuring both baseline and elevated corticosterone levels was chosen. Finally, for mammals, data were restricted to studies that independently measured both cortisol and corticosterone, including both corticosterone‐dominant (> 50% corticosterone) and cortisol‐dominant (> 50% cortisol) species. This filtering process resulted in a dataset of 160 vertebrate species (reptiles, birds, and mammals) that was both taxonomically diverse and broadly representative of the body size range found in each class. This dataset includes a small number of studies using serum corticosterone concentrations as these are indistinguishable from plasma measures (El‐Farhan et al. 2017) (*n* = 4; Table S1). It also includes a small number of subspecies when the original authors considered subspecies (*n* = 3; Table S1).

Given our search criteria, plasma corticosterone concentrations were generally collected from studies aimed at measuring physiological stress. We collected all available data from these papers on baseline and elevated corticosterone concentrations, which represent the measurements taken prior to exposure to a stressor and the measurement taken shortly after exposure to a stressor, respectively. Stressors were categorized into three categories for the purpose of analysis: ACTH injections, restraint methods, or other (e.g., synthetic angiotensin I injection, oxytocin injection, glucocorticoid implant, fasting, transport, and extreme environmental conditions). In studies with multiple measures of hormone levels across individuals, such as by sex or location, concentrations were averaged. If necessary, an online plot digitizer (PlotDigitizer 2025) was used to extract reported values from figures. Finally, from these papers we collected data on a range of potential covariates that were assessed statistically as described below. Covariates considered include sex, season (breeding vs. non‐breeding), environment (captive vs. wild), assay type, and time to bleeding (Table S1).

When available, data on body mass, resting mass‐specific metabolic rate (MSMR), and maximum lifespan were added. Body mass values were taken from the original corticosterone study when possible, and averaged when multiple masses were reported. If masses were unavailable, adult body mass was obtained from an independent source so long as the original study specified the study subjects were adults. Our first choice for an independent source was AnAge (de Magalhães et al. 2024), but if the body mass was not listed there, we used other sources (Table S1). Therefore, studies were largely restricted to adults and only included juveniles when their body mass values were reported in the original study. Resting mass‐specific metabolic rates were taken from AnAge (de Magalhães et al. 2024), or from other sources if not available on AnAge including datasets by Gillooly et al. (2017), White et al. (2005), and Lasiewski and Dawson (1967). All measurements were taken on normothermic, inactive, and conscious individuals. Resting metabolic rates were used here, like in previous studies (Haase et al. 2016; Francis et al. 2018), because these are the only measures of metabolic rate available for such a diverse assortment of species, and because they vary in proportion to active or field metabolic rates across body sizes (Auer et al. 2017; Ricklefs et al. 1996; Savage et al. 2004). Estimates of resting MSMR were restricted to those where the body masses of subjects used to measure MSMR differed by less than twofold from the body masses of subjects from the respective corticosterone study. This was to ensure that both measures of MSMR and corticosterone were taken from similar‐sized individuals, since both measures vary with body mass (Kleiber 1947). In practice, this eliminated just a few MSMR measures from consideration where individuals from large, long‐lived indeterminate growers substantially differed in size between the two studies. Finally, maximum lifespan was estimated using ReptTrait (Oskyrko et al. 2024) for reptiles and AnAge (de Magalhães et al. 2024) for birds and mammals (Table S1).

### 2.2 Statistical Analysis

All relationships were evaluated using phylogenetic generalized least squares (PGLS) regression on natural log‐transformed data to account for evolutionary relatedness among species. In order to test these relationships, a phylogenic tree was constructed for all species included in the study using the Open Tree of Life (Rees and Cranston 2017) through the R package “ape” (Paradis et al. 2019) (Figure S1). Models were built assuming a Brownian motion model of evolution with the R package “nlme” (Pinheiro et al. 2025), and Pagel's *λ* was estimated to test for phylogenetic signal with the R package “phytools” (Revell 2024). Values of Pagel's *λ* range from 0 to 1, where 0 indicates no phylogenetic dependence and values approaching 1 indicate high phylogenetic influence. In the case of erroneously generated lambda values less than zero or greater than one, we took the value to be zero or one, respectively (Revell and Harmon 2022). Likelihood *R* ^2^ values were calculated using the R package “rr2” (Ives 2018) and *p* values were reported for each model.

To account for the possible effects of other abiotic and biotic factors on analyses, we included vertebrate class, sex, season, environment, stressor type, assay, and time to bleed as single categorical additive covariates. These models were then compared in stages using Bayesian information criterion (BIC) (Schwarz 1978). In an effort to account for small sample sizes, the first stage tested the predictive relationship (i.e., baseline corticosterone ~body mass) with vertebrate class added as a covariate, but vertebrate class did not improve model parsimony, so data remained pooled across species for subsequent BIC tests (Table S2). The second stage tested the predictive relationship with assay type and time to bleed as individual covariates to account for method‐based effects. The third and last stage tested the predictive relationship with individual models for sex, breeding season, environment, and stressor type as covariates to account for abiotic environmental or biotic effects. For all models tested in the last two stages, the simplest model without added covariates was either the most parsimonious or only marginally different from the most parsimonious model (∆BIC < 2.3; Table S2). All delta BIC values for predictive relationships fell at or below the threshold of 2 indicating weak support (Kass and Raftery 2012; Raftery 1995) so additive covariates were removed from all further analyses. Furthermore, time to bleed did not significantly affect baseline (Welch's *t*(109.66) = 1.01, *p* = 0.31) or elevated (Welch's *t*(42.83) = −0.68, *p* = 0.50) corticosterone concentration, as samples collected within 3 min of capture did not significantly differ from those collected after 3 min.

For reptiles, the only ectotherms considered here, we also evaluated the possible effects of body temperature on corticosterone levels for the subset of data where body temperature or environmental temperature at the place and time of measurement was given (*n* = 23, range = 18.3°C–32.0°C; Table S1). A likelihood ratio test compared natural log‐transformed models of baseline corticosterone concentrations versus body mass that included temperature as an additive effect, but temperature did not significantly improve the body‐mass dependence of the model (*χ* ^2^ = 0.38, df = 1, *p* = 0.54) and was therefore excluded from further analysis.

The possible effects of corticosterone levels on lifespan were assessed by first plotting natural log‐transformed regressions of maximum lifespan versus baseline corticosterone for reptiles, birds, and mammals. Then, to assess the possible effects of corticosterone on lifespan independent of any effects of MSMR, we plotted the residuals of a natural log‐transformed plot of lifespan versus MSMR against natural log‐transformed baseline corticosterone concentrations for each class.

## 3 Results

**FIGURE 1:**
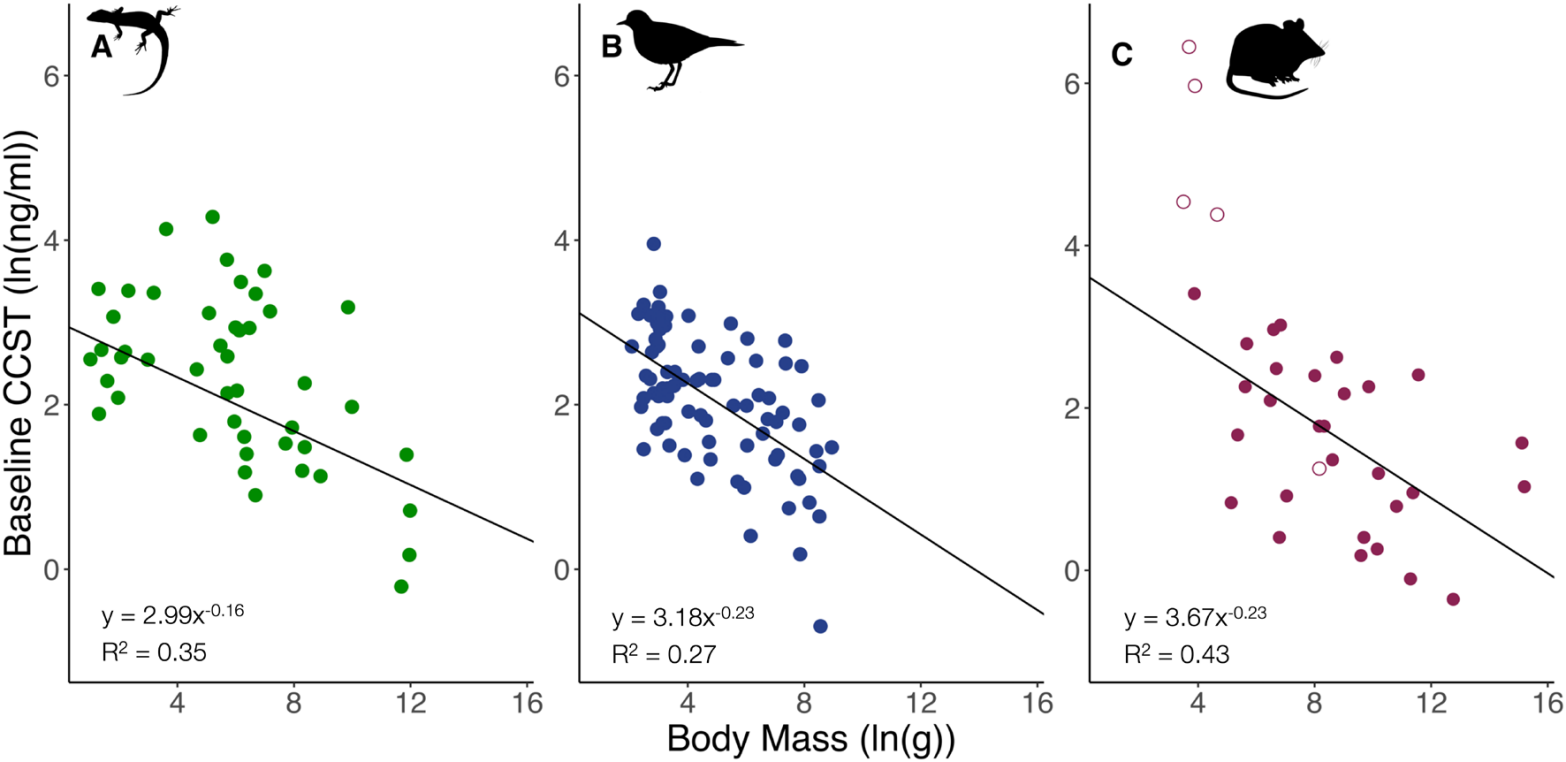
Plots of baseline plasma corticosterone (CCST) concentrations (ng/mL) versus body mass (g) for reptiles (Panel A), birds (Panel B), and mammals (Panel C). For mammals, corticosterone‐ and cortisol‐dominant species are indicated by hollow and solid symbols, respectively. Data are natural log‐transformed, and lines are fit using PGLS regression.

Results generally support the predicted body mass‐dependence of baseline corticosterone concentrations for each of the three vertebrate classes (Equation 1). The slopes of natural log‐transformed plots of baseline corticosterone and body mass are not significantly different than the predicted value of −0.25 for reptiles (−0.16, 95% CI [−0.26, −0.07]), birds (−0.23, 95% CI [−0.30, −0.16]), or mammals (−0.23, 95% CI [−0.41, −0.05]) based on the 95% confidence intervals (Table 1, Figure 1). Between 27% and 43% of the variation in corticosterone levels is explained by body mass (*R* ^2^ = 0.35, 0.27, and 0.43, in reptiles, birds, and mammals, respectively, Table 1).

**TABLE 1:** Results of statistical analyses evaluating relationships of baseline plasma corticosterone (CCST; ng/mL) to body mass (g) and mass‐specific metabolic rate (MSMR; W/g) in reptiles, birds, and mammals. Statistical analysis of the relationship between elevated and baseline corticosterone levels across all vertebrate classes is also presented.

	*n*	Predicted slope	Slope (95% CI)	Intercept	*R* ^2^	*p*	*λ*
**Reptiles**							
Baseline CCST vs. body mass	46	−0.25	−0.16 (−0.26, −0.07)	2.99	0.35	**< 0.001**	0.61
Baseline CCST vs. MSMR	23	1	0.28 (0.02, 0.54)	4.20	0.17	**0.04**	0.42
**Birds**							
Baseline CCST vs. body mass	81	−0.25	−0.23 (−0.30, −0.16)	3.18	0.27	**< 0.001**	0
Baseline CCST vs. MSMR	30	1	0.85 (0.55, 1.15)	6.03	0.47	**< 0.001**	0
**Mammals**							
Baseline CCST vs. body mass	34	−0.25	−0.23 (−0.41, −0.05)	3.67	0.43	**0.01**	0.73
Baseline CCST vs. MSMR	25	1	1.36 (0.81, 1.91)	10.43	0.49	**< 0.001**	0
**All vertebrates**							
Elevated CCST vs. baseline CCST	109		0.69 (0.57, 0.82)	2.07	0.49	**< 0.001**	0.19

*Note:* Bold values indicate statistically significant *p* vlaues (*p* < 0.05).

**FIGURE 2:**
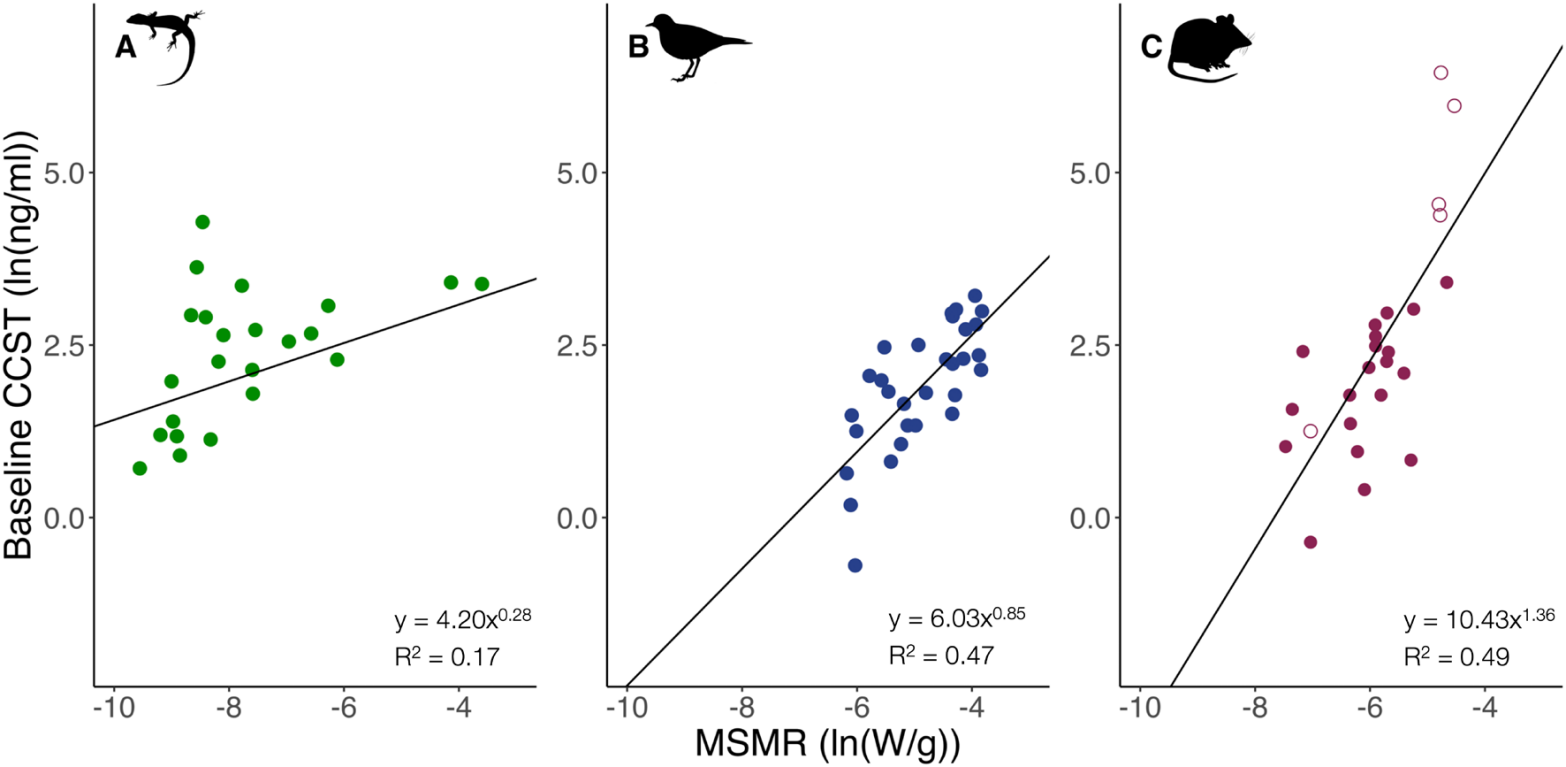
Plots of baseline plasma corticosterone (CCST) concentrations (ng/mL) versus mass‐specific metabolic rate (MSMR, W/g) for reptiles (Panel A), birds (Panel B), and mammals (Panel C). For mammals, corticosterone‐ and cortisol‐dominant species are indicated by hollow and solid symbols, respectively. Data are natural log‐transformed, and lines are fit using PGLS regression.

**FIGURE 3:**
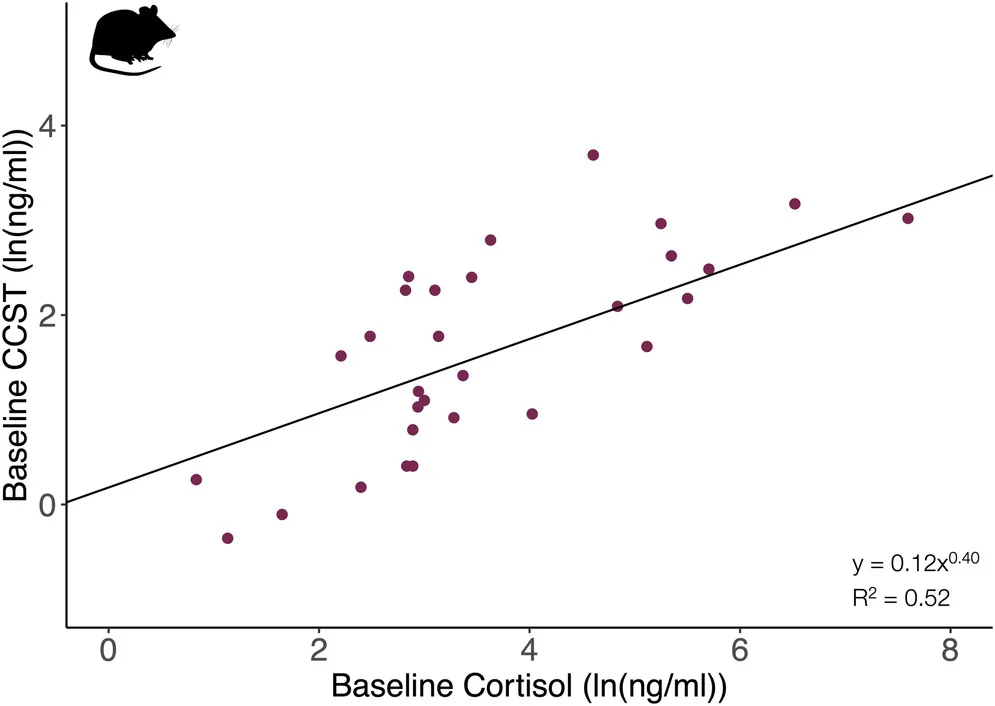
Plot of baseline corticosterone (CCST) concentrations (ng/mL) versus baseline cortisol concentrations (ng/mL) for cortisol‐dominant mammals. Data are natural log‐transformed, and lines are fit using PGLS regression.

The results also generally support the predicted dependence of corticosterone concentrations on metabolic rate (Equation 1). In birds and mammals, natural log‐transformed plots of baseline corticosterone concentrations versus MSMR show statistically significant relationships with slopes that do not significantly differ from the predicted slope of 1 (0.85, 95% CI [0.55, 1.15], 1.36, 95% CI [0.81, 1.91], respectively, Table 1, Figure 2). In birds and mammals, 47% and 49% of the variation in corticosterone levels, respectively, are described by metabolic rate with no phylogenetic influence on the relationships (*λ* = 0, Table 1). Reptiles also show a statistically significant relationship between baseline corticosterone concentrations and MSMR, but the slope is significantly less than 1 (0.28, 95% CI [0.02, 0.54], Table 1) and the relationship is strongly influenced by two species with high metabolic rates (Figure 2A). We see no relationship between corticosterone and mass‐specific metabolic rate if we exclude these two points. In the case of mammals, baseline corticosterone concentrations show a highly significant, hypoallometric relationship with cortisol concentrations across cortisol‐dominant species (slope = 0.40, 95% CI [0.21, 0.59], *p* < 0.001, Figure 3).

**FIGURE 4:**
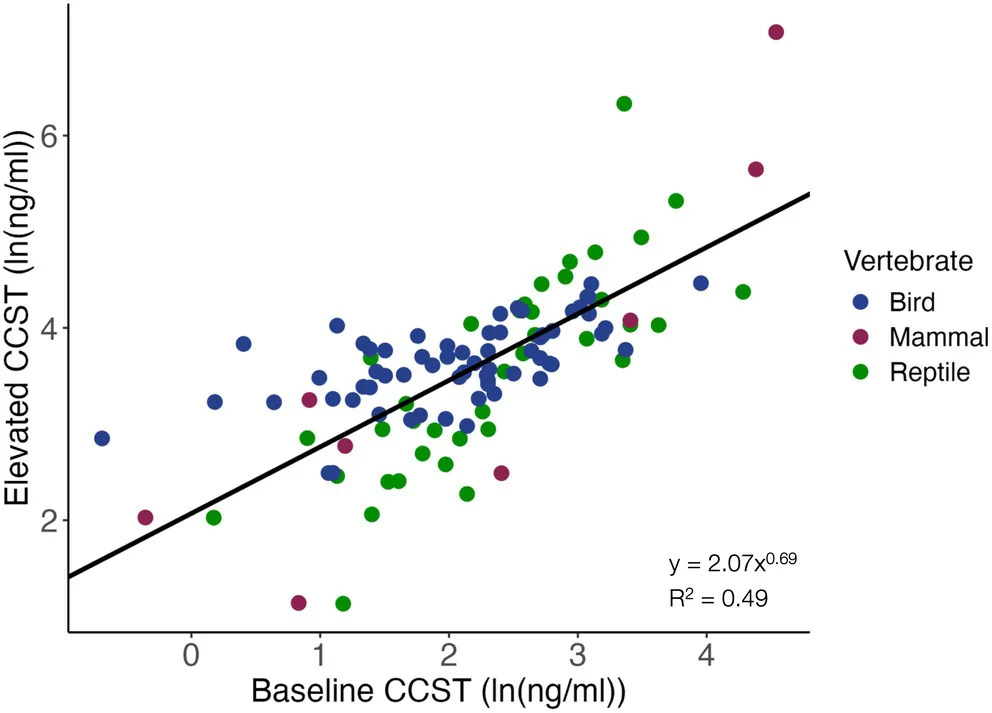
Plot of elevated plasma corticosterone (CCST) concentrations (ng/mL) versus baseline plasma corticosterone concentrations (ng/mL) across reptiles, birds, and mammals. Data are natural log‐transformed, and lines are fitted using PGLS regression.

Moreover, across all vertebrates, the results show a strong, positive relationship between elevated and baseline corticosterone concentrations (0.69, 95% CI [0.57, 0.82], Table 1, Figure 4). As alluded to in the methods, stressor type did not significantly improve model parsimony when added as a covariate (∆BIC > 2; Table S2). *R* ^2^ values indicate almost 50% of the variation in elevated concentrations is explained by baseline concentrations (*R* ^2^ = 0.49, Table 1). Within classes, reptiles and mammals show stronger correlations that did not significantly differ from unity whereas birds show a weaker correlation with a predicted slope that is significantly less than one (reptiles = 0.97, 95% CI [0.71, 1.23], *R* ^2^ = 0.58, birds = 0.36 95% CI [0.26, 0.47], *R* ^2^ = 0.36, mammals = 0.95 95% CI [0.41, 1.48], *R* ^2^ = 0.70; all = *p* < 0.01).

With respect to lifespan, across all three classes of vertebrates, we initially observed a strong, inverse relationship between lifespan and baseline corticosterone levels (*p* < 0.01, reptiles = −0.46, 95% CI [−0.71, −0.21], birds = −0.37, 95% CI [−0.55, −0.19], mammals = −0.22, 95% CI [−0.43, −0.01]; Figure S2A–C). However, after removing the effects of metabolic rate on this relationship through residual analysis, we find no statistically significant relationship between lifespan and corticosterone (*p* > 0.05, reptiles = −0.02, 95% CI [−0.31, 0.27], birds = −0.02, 95% CI [−0.21, 0.16], mammals = 0.04, 95% CI [−0.17, 0.26], Figure S2D–F).

## 4 Discussion

The relationships between corticosterone and metabolic rate shown here raise important questions regarding the interpretation of GC levels across species as measures of physiological stress. Results show that baseline corticosterone concentrations increase in proportion to mass‐specific metabolic rate in birds and mammals, with the relationship being weaker and less than linear in reptiles (Figure 2). Still, all three classes (reptiles, birds, and mammals) show the predicted body mass‐dependence based on the scaling of mass‐specific metabolic rate (Equation 1, Figure 1). Note, too, that the relationship is remarkably similar between birds, which possess only corticosterone, and mammals, which possess both cortisol and corticosterone (Figure 1). Therefore, for a given body mass, birds and mammals have similar baseline corticosterone levels. Our results for corticosterone across these three classes are similar to the relationships observed between cortisol, mass‐specific metabolic rate, and body mass in cortisol‐dominant mammals (Haase et al. 2016), and to those describing the relationship of fecal glucocorticoids to mass‐specific metabolic rate and body mass in mammals (Hudson et al. 2026). They are also similar to a previously reported relationship between glucocorticoids (cortisol or corticosterone) and body mass across vertebrates (Francis et al. 2018).

However, our results differ from those found by Francis et al. (2018) within these vertebrate classes. They found no relationship between glucocorticoid concentration and mass‐specific metabolic rate in each of these three classes, and only found the predicted relationship with body mass in mammals. This may be due to a few factors. First, for example, the data compilation from Francis et al.'s (2018) study consisted exclusively of field studies, a large portion of which were originally focused on the endocrinology of reproduction (e.g., courtship, pregnancy, etc.), especially for birds. In contrast, our study focused on compiling both captive and field studies aimed at testing the stress response and included few breeding individuals. Cortisol and corticosterone are highly variable during breeding season (Michael Romero 2002; Wingfield et al. 1998). Second, with respect to mammals, Francis et al. (2018) used measures of only a single GC hormone (cortisol or corticosterone) for each species and then combined them into a single category (i.e., glucocorticoids) for analysis. In contrast, we focused only on the relationship of corticosterone to mass‐specific metabolic rate and body mass. Strictly speaking, then, these analyses for mammals are not comparable. Third, for reptiles, Francis et al. (2018) made the reasonable assumption that GC levels vary with temperature and thus estimated reptile body temperature at the time of measurement using the average seasonal temperature at the location of the study (Francis et al. 2018; Johnson et al. 2018). They then plotted the residuals from a plot of GC levels versus average seasonal temperature against body mass or temperature‐adjusted metabolic rate to remove the effects of temperature in assessing relationships of reptile GC levels to these variables (Francis et al. 2018). In contrast, our estimates of temperatures consisted of environmental temperatures at the time and place of corticosterone measurement. Our analysis of these data found no effect of temperature on GC levels, so subsequent analyses did not adjust corticosterone for temperature. Here again, then, our results and those of Francis et al. (2018) are not strictly comparable.

The relationship we show between corticosterone and mass‐specific metabolic rate might reasonably lead one to expect that corticosterone levels should increase exponentially with temperature in the same way as metabolic rate (Gillooly et al. 2001; Brown et al. 2004). Yet, we did not observe this temperature dependence among reptiles. We attribute this to the relatively narrow range of environmental temperatures reported in these studies, most of which fell between 20°C–30°C, the relatively small subset of studies that reported temperature, and the possibility that core body temperatures differ significantly from environmental temperature given the propensity of reptiles to behaviorally thermoregulate. Nevertheless, we think it's reasonable to broadly expect that corticosterone levels in ectotherms are likely to increase by two to threefold for every 10°C increase in temperature in the same way as metabolic rate. At present, many studies do not even report temperature, so the possibility that environmental temperature may strongly influence corticosterone levels in ectotherms is underappreciated. The few studies that have explored a temperature‐dependence of glucocorticoid levels support this expectation (Rubalcaba and Jimeno 2022; Tyrrell and Cree 1998; Jessop et al. 2016).

Still, our results provide support for the notion that physiological stress, based on measures of cortisol and/or corticosterone, has a large metabolic component (Jimeno et al. 2018; Jimeno and Verhulst 2023). To the extent that this metabolic component of stress is the dominant component, physiological stress could perhaps be described as an energetic rate process where a higher‐than‐expected metabolic rate for a given body mass and/or temperature could be viewed as a measure of stress. Among vertebrates, stress is not typically thought of as a rate process, much less an energetic rate process, but among invertebrates, researchers have used changes in rates of energy expenditure as a measure of physiological stress (Hawlena and Schmitz 2010). In which case, models describing the body mass‐ and temperature‐dependence of metabolic rate for different classes of vertebrates might provide a point of departure for assessing stress levels among species (Kleiber 1947; Gillooly et al. 2001). At the very least, our results indicate that comparisons of physiological stress based on corticosterone levels must account for differences in body mass and/or metabolic rate among species. Otherwise, one might falsely conclude that an individual or species with a higher metabolic rate is more stressed.

The relationship between corticosterone levels and metabolic rate is also useful for understanding the magnitude of physiological stress, or the difference between baseline and elevated corticosterone levels (Figure 4). On average, elevated corticosterone levels were 4.2‐fold higher than baseline levels at an intermediate baseline level (i.e., 7 ng/mL). Moreover, for reptiles and mammals, the scaling exponent for these relationships is not significantly different than one indicating that the magnitude of the stress response is reasonably constant across body sizes. These results are remarkably consistent with previous work in mammals showing that elevated cortisol levels in plasma and feces increase in proportion to baseline levels (Haase et al. 2016; Hudson et al. 2026) by 3.5‐fold and 3.4‐fold, respectively. Interestingly, Haase et al. (2016) attribute this difference to the approximately 3.5‐fold difference between active and maximal metabolic rates of mammals (Haase et al. 2016; Savage et al. 2004), thus suggesting that the magnitude of the stress response is also strongly influenced by energetics.

The relationship between corticosterone levels and metabolic rate may also provide insights into how and why chronic stress levels impact lifespan. We found that the negative relationship between corticosterone levels and lifespan was largely due to the relationship between corticosterone and metabolic rate. In other words, we found no independent effect of corticosterone on lifespan after removing the effects of metabolic rate. This suggests that the metabolic component of stress, and associated free radical production, may be responsible for the detrimental effects of chronic stress (Aschbacher et al. 2013; Yegorov et al. 2020; Pickering et al. 2013). The damage from chronic stress is cumulative (Selye 1975), which implies that stress is a rate process not unlike metabolic rate or free radical production rate. Chronic stress thus accelerates aging, or stated another way, accelerates the rate of living. There is a vast literature linking the rate of living to the rate of energy use and free radical production across species, beginning with Raymond Pearl's Rate of Living Theory (Beckman and Ames 1998; Harman 1956, 1992; Pearl 1928). Further considering how this body of work relates to physiological stress may be helpful in developing models that predict how the intensity or duration of stress episodes impact lifespan. That said, we recognize that GCs may impact health or longevity in a variety of ways, apart from those related to metabolic rate (Sapolsky et al. 2000; Cidlowski and Whirledge 2013; McEwen and Stellar 1993). We also recognize the accumulation of oxidative damage is not simply a function of free radical production. Species may evolve mechanisms to mitigate damage caused by free radicals and extend lifespan (Selman et al. 2012). Moreover, species presumably may evolve to optimize GC levels to match their life histories (Sapolsky 2021). So, for example, a larger long‐lived species may have evolved to have relatively low GC levels to minimize oxidative damage.

However, we acknowledge that there is a significant amount of unexplained variation in the relationships shown here. Results show that 50% or more of the variation in corticosterone across species was not explained by metabolic rate or body size (Figures 1 and 2). In part, this variation is attributed to the lack of standardization in measuring baseline or elevated levels across species (Romero et al. 2015; Moberg 1987). In practice, baseline levels represent the measure prior to a stressor being applied rather than a measure at a defined physiological state or condition (Moberg 1987). Unlike basal metabolic rate, measures of baseline corticosterone levels may be measured at different activity levels or digestive states, and in different environmental conditions (MacDougall‐Shackleton et al. 2019). Similarly, elevated levels are not necessarily peak levels and often are not measured at standardized times after the stressor is applied (Moberg 1987). We speculate that the time to peak stress level may itself be related to body size and metabolic rate; though, at present, sufficient data are not available to assess this hypothesis. Finally, it is important to note that hormone concentration alone does not determine the overall stress response (MacDougall‐Shackleton et al. 2019). For example, new world primates possess low‐affinity GC receptors that render them resistant to high levels of glucocorticoids (Chrousos et al. 1982). There are, of course, many other dimensions of physiological stress beyond the metabolic component, including cognitive changes, behavioral shifts, and gene expression levels (de Kloet et al. 2005; la Fleur 2006; Juszczak and Stankiewicz 2018).

Nevertheless, it is our hope that quantifying variation in corticosterone levels across species and relating it to metabolic rate may provide a step toward developing a more general comparative framework. This framework could prove useful for evaluating the detrimental effects of chronic stress on key aspects of individual fitness related to energetics.

## Author Contributions

**Alyson R. Wisnionski:** data curation (lead), formal analysis (lead), investigation (supporting), methodology (equal), project administration (lead), visualization (lead), writing – original draft (lead), writing – review and editing (lead). **Catherine G. Haase:** conceptualization (supporting), data curation (supporting), investigation (equal), writing – review and editing (supporting). **Isabella Dy:** investigation (equal), writing – review and editing (supporting). **Kyle Hudson:** formal analysis (supporting), methodology (supporting), writing – review and editing (supporting). **James F. Gillooly:** conceptualization (lead), methodology (equal), project administration (supporting), supervision (lead), visualization (supporting), writing – review and editing (equal).

## Funding

The authors have nothing to report.

## Conflicts of Interest

The authors declare no conflicts of interest.

## Supporting information


**Figure S1:** Phylogenetic tree of all reptiles, birds, and mammals included in data analyses.
**Figure S2:** Plots to evaluate effects of corticosterone on lifespan for reptiles, birds, and mammals.
**Table S1:** Data include body mass (Mass; g), mass‐specific metabolic rate (MSMR; mW/g), lifespan (LS; years), baseline/elevated plasma corticosterone concentrations (CCST; ng/mL), assay type (RIA = radioimmunoassay, EIA = enzyme immunoassay, ELISA = enzyme‐linked immunosorbent assay, DIDA = double isotope dilution assay), time to bleed (Time; min), and stressor type for reptiles, birds, and mammals used in this study.
**Table S2:** Results for model selection using Bayesian information criterion (BIC).

## Data Availability

Data used in the current study are provided in the Supporting Information. All data, R scripts, and associated files used in this study are publicly available in Dryad Digital Repository: https://doi.org/10.5061/dryad.79cnp5jbt.
