# Supplementary material for: Corticosterone Concentration Varies With Metabolic Rate in Vertebrates
Source: Ecol Evol. 2026 Aug 27;16(9):e74243. doi: 10.1002/ece3.74243 (PMC13519521; doi:10.1002/ece3.74243)
Supplement: Supplementary file 1 [file ECE3-16-e74243-s001.docx]

**Supplementary Materials for “Corticosterone Concentration Varies with Metabolic Rate in Vertebrates”**

Alyson R. Wisnionski^1*^, Catherine G. Haase^2^, Isabella Dy^1^, Kyle Hudson^1^, & James F. Gillooly^1^

^1^Department of Biology, University of Florida, Gainesville, FL 32611, USA

^2^Department of Biology, Austin Peay State University, Clarksville, TN 37044, USA

**Author for correspondence*: awisnionski@ufl.edu

The following supplementary materials for “Corticosterone Concentration Varies with Metabolic Rate in Vertebrates” contain all data and associated references (Table S1), the phylogenetic tree of all reptiles, birds, and mammals included in analyses (Figure S1), BIC model selection criteria (Table S2), and plots showing the relationships between lifespan and corticosterone both before and after accounting for the effects of mass-specific metabolic rate (Figure S2).

**Table S1:** Data include body mass (Mass; g), mass-specific metabolic rate (MSMR; mW/g), lifespan (LS; years), baseline/elevated plasma corticosterone concentrations (CCST; ng/ml), assay type (RIA = radioimmunoassay, EIA = enzyme immunoassay, ELISA = enzyme-linked immunosorbent assay, DIDA = double isotope dilution assay), time to bleed (Time; min), and stressor type for reptiles, birds, and mammals used in this study. Sample size (N), sex (M = males, F = females, MF = both), season (B = breeding, NB = non-breeding), environment (C = captive, F = field), and baseline plasma cortisol concentrations (ng/ml) are also included, with “U” indicating information unspecified. Temperature (Temp; °C) is included for reptiles. Asterisks (*) indicate serum glucocorticoid hormone concentrations and carrot symbols (^) indicate juveniles.

| Species | Mass  (g) | |  | MSMR  (mW/g) |  | LS  (yrs) |  | Temp (°C) | N | Sex | Season | Envr | Baseline CCST  (ng/ml) | Elevated CCST  (ng/ml) | Baseline cortisol  (ng/ml) | Time (min) | Stressor^1^ | Assay |  |
| --- | --- | --- | --- | --- | --- | --- | --- | --- | --- | --- | --- | --- | --- | --- | --- | --- | --- | --- | --- |
| Reptiles |  | |  |  |  |  |  |  |  |  |  |  |  |  |  |  |  |  |  |
| *Agkistrodon piscivorus* | 421 | | [1] |  |  | 24.6 | [2] | - | 32 | MF | U | F | 8.77 | 56.97 |  | >3 | Restraint | EIA | [1] |
| *Alligator mississippiensis^* | 793 | | [3] | 0.14 | [4] | 85.0 | [2] | - | 10 | U | U | C | 2.46 | 17.35 |  |  | Restraint | RIA | [3] |
| *Amblyrhynchus cristatus* | 2227 | | [4] |  |  | 28.0 | [2] | 20.5 | 98 | U | U | F | 4.61 | 11.02 |  | <3 | Restraint | RIA | [5] |
| *Anolis carolinensis* | 3 | | [6] | 0.95 | [7] | 11.0 | [2] | 20.0 | 42 | F | U | C | 12.83 | - |  | <3 | - | RIA | [6] |
| *Anolis sagrei* | 5 | | [8] | 2.20 | [9] | 8.0 | [2] | - | 114 | M | U | F | 9.86 | - |  | >3 | - | RIA | [8] |
| *Antaresia childreni* | 384 | | [4] | 0.51 | [7] | 30.0 | [2] | 29.6 | 16 | F | NB | C | 6.02 | 14.78 |  |  | Restraint | RIA | [10] |
| *Aspidoscelis sexlineatus* | 8 | | [4] |  |  |  |  | 32.0 | 35 | MF | U | C | 13.13 | 41.85 |  | >3 | Restraint | RIA | [11] |
| *Aspidoscelis uniparens* | 7 | | [12] |  |  |  |  | 32.0 | 17 | U | U | C | 8.04 | 17.26 |  | >3 | Restraint | RIA | [12] |
| *Boa constrictor* | 3950 | | [13] | 0.10 | [4] | 40.4 | [2] | - | 15 | MF | U | C | 3.31 | - |  |  | - | EIA | [13] |
| *Boaedon fuliginosus* | 182 | | [14] | 0.21 | [15] |  |  | 27.0 | 24 | MF | U | C | 72.44 | 79.43 |  | >3 | Other | ELISA | [14] |
| *Caiman latirostris^* | 161 | | [16] |  |  | 22.0 | [2] | 31.4 | 24 | U | U | C | 22.50 | - |  |  | - | RIA | [16] |
| *Caretta caretta* | 156500 | | [4] |  |  | 76.0 | [2] | 28.1 | 46 | U | U | F | 1.19 | 7.59 |  |  | Restraint | RIA | [17] |
| *Chelonia mydas* | 160000 | | [4] | 0.07 | [18] | 75.0 | [2] | - | 20 | MF | U | F | 2.04 | - | 1.78 |  | - | ELISA | [19] |
| *Chelonoidis niger* | 118535 | | [20] |  |  | 177.0 | [2] | - | 16 | MF | U | C | 0.81 | - |  | >3 | - | RIA | [21] |
| *Crocodylus johnstoni* | 141832 | | [22] | 0.13 | [23] | 20.0 | [2] | - | 44 | MF | U | F | 4.02 | 40.02 |  | >3 | Restraint | RIA | [22] |
| *Crocodylus niloticus^* | 540 | | [24] |  |  | 80.0 | [2] | 30.0 | 5 | U | U | C | 5.00 | 11.10 |  |  | Other | RIA | [24] |
| *Crotalus atrox* | 480 | | [25] |  |  | 27.0 | [2] | - | 12 | M | U | C | 32.87 | 140.00 |  | <3 | Restraint | RIA | [25] |
| *Crotalus viridis* | 800 | | [26] |  |  | 31.0 | [2] | - | 30 | M | U | F | 28.44 | 39.01 |  | >3 | Other | RIA | [26] |
| *Elgaria coerulea* | 20 | | [27] |  |  | 8.0 | [2] | 23.5 | 15 | MF | U | C | 12.77 | 65.46 |  | <3 | ACTH | RIA | [27] |
| *Elgaria multicarinata* | 24 | | [27] | 0.42 | [4] | 15.0 | [2] | 23.5 | 15 | MF | U | C | 28.80 | 561.60 |  | <3 | ACTH | RIA | [27] |
| *Gopherus agassizii* | 2784 | | [4] |  |  | 100.0 | [2] | - | 11 | MF | U | C | 5.60 | 20.72 |  |  | ACTH | RIA | [28] |
| *Heloderma horridum* | 586 | | [29] |  |  |  |  | 30.0 | 7 | M | U | C | 4.06 | 7.85 |  | <3 | Restraint | ELISA | [29] |
| *Heloderma suspectum* | 553 | | [4] | 0.14 | [30] | 48.0 | [2] | 30.0 | 8 | MF | U | C | 3.25 | 3.10 |  | <3 | Restraint | ELISA | [31] |
| *Hoplodactylus duvaucelii* | 118 | | [4] |  |  | 50.0 | [2] | 18.3 | 16 | MF | NB | C | 5.11 | - |  | >3 | - | RIA | [32] |
| *Lepidochelys kempii^* | 4300 | | [33] |  |  | 50.0 | [2] | 25.2 | 18 | U | U | C | 4.41 | 19.02 |  | >3 | Other | RIA | [33] |
| *Liopholis whitii* |  | |  |  |  |  |  | - | 14 | M | NB | F | 5.30 | 24.80 |  | >3 | Restraint | RIA | [34] |
| *Malayopython reticulatus* | 21750 | | [35] | 0.12 | [36] | 29.4 | [2] | 28.5 | 11 | MF | U | F | 7.20 | 13.20 |  | <3 | Other | ELISA | [35] |
| *Natrix tessellata* | 303 | | [37] |  |  | 14.0 | [2] | - | 75 | MF | B | F | 13.30 | 69.78 |  | >3 | Restraint | RIA | [37] |
| *Nerodia rhombifer* | 238 | | [7] | 0.53 | [7] | 10.0 | [2] | - | 12 | MF | NB | F | 15.16 | 86.10 |  | >3 | Restraint | ELISA | [38] |
| *Nerodia sipedon* | 1306 | | [4] |  |  | 21.0 | [2] | - | 25 | MF | NB | F | 22.99 | 119.86 |  | >3 | Restraint | ELISA | [38] |
| *Phrynocephalus vlangalii* |  | |  |  |  | 6.0 | [2] | - | 11 | MF | U | C | 10.02 | 19.03 |  | <3 | Other | EIA | [39] |
| *Pituophis c. affinis* | 457 | | [29] | 0.22 | [40] | 33.8 | [2] | 30.0 | 4 | U | U | C | 18.24 | 93.04 |  | <3 | Restraint | ELISA | [29] |
| *Podarcis siculus* | 9 | | [7] | 0.30 | [7] |  |  | - | 20 | M | NB | F | 14.06 | 64.46 |  | >3 | Restraint | RIA | [41] |
| *Python bivittatus* | 19050 | | [42] |  |  | 28.3 | [2] | - | U | U | B | F | 24.16 | 73.20 |  | >3 | Restraint | EIA | [42] |
| *Python regius* | 1086 | | [43] | 0.19 | [7] | 50.0 | [2] | 29.0 | 4 | U | U | C | 37.60 | 56.20 |  |  | Restraint | RIA | [43] |
| *Regina septemvittata* | 298 | | [4] |  |  | 19.3 | [2] | - | 15 | MF | NB | F | 43.05 | 204.50 |  | >3 | Restraint | ELISA | [38] |
| *Sceloporus occidentalis* | 10 | | [40] | 27.36 | [40] | 7.0 | [2] | 30.0 | 13 | U | U | C | 29.53 | - |  | <3 | - | RIA | [44] |
| *Sceloporus undulatus* | 6 | | [45] | 1.88 | [7] | 4.0 | [2] | - | 41 | MF | U | C | 21.52 | 48.78 |  | <3 | Restraint | EIA | [45] |
| *Sphenodon punctatus* | 645 | | [46] | 0.17 | [7] | 100.0 | [2] | - | 5 | M | U | F | 18.78 | - |  |  | - | RIA | [46] |
| *Thamnophis errans* | 400 | | [4] |  |  | 17.8 | [2] | - | 51 | M | U | F | 18.89 | 108.67 |  | <3 | ACTH | RIA | [47] |
| *Thamnophis s. parietalis* | 37 | | [48] |  |  | 20.0 | [2] | - | 10 | M | U | F | 62.50 | - |  |  | - | RIA | [48] |
| *Tiliqua scincoides* | 303 | | [43] | 0.50 | [7] | 30.0 | [2] | 29.0 | 3 | M | U | C | 8.50 | 9.71 |  |  | Restraint | RIA | [43] |
| *Urosaurus ornatus* | 4 | | [49] |  |  | 3.0 | [2] | - | 41 | MF | U | F | 6.61 | 18.81 |  | >3 | Restraint | RIA | [50] |
| *Uta stansburiana* | 4 | | [40] | 15.96 | [40] | 4.8 | [2] | - | 173 | F | U | F | 30.20 | 56.50 |  | <3 | Restraint | RIA | [51] |
| *Varanus salvator* | 7400 | | [35] | 0.24 | [52] | 15.8 | [2] | 28.5 | 10 | MF | B | F | 3.10 | 11.70 |  | <3 | Other | ELISA | [35] |
| *Varanus varius* | 4300 | | [4] | 0.28 | [7] | 22.0 | [2] | - | 12 | U | U | F | 9.59 | 22.87 |  | >3 | Restraint | RIA | [53] |
| *Vipera aspis* | 106 | | [54] |  |  | 25.0 | [2] | 24.0 | 33 | MF | NB | C | 11.35 | 34.70 |  | >3 | Other | RIA | [54] |
| *Zootoca vivipara* | 4 | | [55] | 1.40 | [40] | 12.0 | [2] | 24.5 | 17 | F | NB | C | 14.40 | 50.80 |  | >3 | Restraint | RIA | [55] |
| Birds |  | |  |  |  |  |  |  |  |  |  |  |  |  |  |  |  |  |  |
| *Acanthis flammea* | 13 | | [56] | 20.63 | [4] | 12.2 | [4] |  | 78 | MF | B | F | 10.50 | 27.50 |  | <3 | Restraint | RIA | [57] |
| *Aethia pusilla* | 85 | | [58] |  |  | 8.1 | [4] |  | 161 | U | B | F | 6.50 | 37.00 |  | <3 | Restraint | RIA | [58] |
| *Aimophila carpalis* | 16 | | [59] |  |  |  |  |  | 41 | M | B | F | 14.00 | 43.03 |  | <3 | Restraint | ELISA | [59] |
| *Amazona ventralis** | 239 | | [60] |  |  |  |  |  | 40 | U | U | C | 19.80 | - |  | <3 | - | RIA | [60] |
| *Anas platyrhynchos^* | 2709 | | [61] | 3.99 | [4] | 29.1 | [4] |  | 33 | MF | U | C | 11.80 | - |  | <3 | - | RIA | [61] |
| *Antigone canadensis** | 5000 | | [62] | 2.07 | [63] | 36.6 | [4] |  | 8 | MF | U | F | 1.90 | 25.20 |  |  | ACTH | RIA | [62] |
| *Aphelocoma coerulescens* | 76 | | [64] |  |  | 15.0 | [4] |  | 35 | MF | U | F | 3.00 | 26.10 |  | <3 | Restraint | RIA | [64] |
| *Aphrastura spinicauda* | 12 | | [65] |  |  |  |  |  | 155 | MF | B | F | 8.00 | 32.74 |  | <3 | Restraint | RIA | [65] |
| *Asio otus* | 262 | | [56] | 3.79 | [4] | 27.8 | [4] |  | 24 | MF | NB | F | 7.30 | 40.40 |  | <3 | Restraint | RIA | [66] |
| *Calcarius lapponicus* | 27 | | [56] |  |  | 6.0 | [4] |  | 12 | MF | B | F | 11.00 | 63.24 |  | <3 | Restraint | RIA | [67] |
| *Calcarius mccownii* | 25 | | [68] |  |  |  |  |  | 33 | MF | B | C | 5.90 | 22.00 |  | <3 | Restraint | RIA | [69] |
| *Calcarius ornatus* | 19 | | [56] |  |  |  |  |  | 45 | MF | B | F | 5.50 | 21.00 |  | <3 | Restraint | RIA | [69] |
| *Calcarius pictus* | 27 | | [4] |  |  | 5.0 | [4] |  | 163 | MF | B | F | 8.20 | 42.20 |  | <3 | Restraint | RIA | [70] |
| *Calidris canutus* | 135 | | [71] |  |  | 27.3 | [4] |  | U | U | U | F | 10.00 | - |  | >3 | - | RIA | [71] |
| *Calidris melanotos* | 81 | | [68] |  |  |  |  |  | 17 | MF | B | F | 10.10 | 51.82 |  | <3 | Restraint | RIA | [72] |
| *Calidris pusilla* | 26 | | [68] |  |  | 19.3 | [4] |  | 52 | MF | B | F | 21.60 | 75.46 |  | <3 | Restraint | RIA | [72] |
| *Columba livia* | 300 | | [73] | 5.33 | [63] | 12.6 | [4] |  | 15 | U | B | F | 2.90 | 12.07 |  | <3 | Restraint | RIA | [73] |
| *Cuculus canorus* | 102 | | [74] | 8.22 | [4] | 12.9 | [4] |  | 25 | MF | B | F | 6.10 | - |  | >3 | - | EIA | [74] |
| *Cuculus poliocephalus* | 56 | | [74] |  |  |  |  |  | 21 | MF | B | F | 6.80 | - |  | >3 | - | EIA | [74] |
| *Cyanistes caeruleus* | 11 | | [68] |  |  | 14.6 | [4] |  | U | MF | B | F | 7.20 | 21.20 |  | <3 | Restraint | RIA | [68] |
| *Daption capense* | 472 | | [75] |  |  | 25.0 | [4] |  | 27 | MF | B | F | 1.50 | 46.20 |  |  | Restraint | RIA | [75] |
| *Diomedea exulans* | 7650 | | [56] | 2.26 | [7] | 50.0 | [4] |  | 75 | MF | B | F | 4.35 | - |  |  | - | RIA | [75] |
| *Empidonax oberholseri* | 21 | | [68] |  |  | 9.0 | [4] |  | U | MF | B | F | 29.10 | 43.45 |  | <3 | Restraint | RIA | [68] |
| *Eudyptes chrysocome* | 2500 | | [56] |  |  |  |  |  | 7 | U | B | F | 5.80 | 50.19 |  | <3 | Restraint | RIA | [76] |
| *Ficedula hypoleuca* | 12 | | [77] | 19.33 | [4] | 15.0 | [4] |  | U | MF | B | F | 24.90 | 54.52 |  | <3 | Other | RIA | [77] |
| *Gallus gallus* | 2580 | | [4] | 2.22 | [7] |  |  |  | 6 | F | U | C | 1.20 | 25.26 |  |  | ACTH | EIA | [78] |
| *Haemorhous mexicanus* | 23 | | [79] | 13.64 | [4] | 11.6 | [4] |  | 6 | M | B | F | 5.90 | - |  | >3 | - | RIA | [79] |
| *Histrionicus histrionicus* | 623 | | [56] |  |  | 14.6 | [4] |  | 38 | MF | B | F | 8.30 | 34.40 |  | <3 | Restraint | RIA | [80] |
| *Horornis diphone* | 17 | | [68] |  |  |  |  |  | U | M | B | F | 52.20 | 86.90 |  | <3 | Restraint | RIA | [68] |
| *Junco hyemalis* | 20 | | [56] | 16.40 | [4] | 11.3 | [4] |  | 40 | MF | B | F | 15.30 | 50.71 |  | <3 | Restraint | RIA | [81] |
| *Kieneria aberti* | 46 | | [56] | 15.76 | [4] | 8.6 | [4] |  | 31 | M | B | F | 10.00 | 42.91 |  | <3 | Restraint | RIA | [82] |
| *Lagopus muta* | 422 | | [56] |  |  |  |  |  | 9 | MF | U | F | 16.50 | 52.89 |  | <3 | Restraint | RIA | [83] |
| *Larus hyperboreus* | 1576 | | [75] | 7.21 | [63] | 21.1 | [4] |  | 38 | MF | B | F | 12.20 | 33.95 |  |  | Restraint | RIA | [75] |
| *Leuconotopicus borealis* | 44 | | [56] |  |  |  |  |  | 113 | MF | B | F | 10.00 | 30.42 |  | <3 | Restraint | RIA | [84] |
| *Limosa lapponica* | 216 | | [85] |  |  | 34.0 | [4] |  | U | MF | NB | F | 13.00 | 65.30 |  | >3 | Restraint | RIA | [85] |
| *Megadyptes antipodes* | 5200 | | [68] | 2.40 | [86] |  |  |  | 16 | MF | B | F | 0.50 | 17.30 |  | >3 | Restraint | EIA | [87] |
| *Melanitta deglandi* | 1194 | | [88] |  |  |  |  |  | 12 | MF | U | C | 4.00 | 43.75 |  | <3 | ACTH | EIA | [88] |
| *Melospiza melodia* | 21 | | [56] | 13.09 | [4] | 11.3 | [4] |  | U | MF | B | F | 18.50 | - |  | >3 | - | RIA | [89] |
| *Mimus polyglottos* | 49 | | [68] |  |  | 20.0 | [4] |  | U | MF | B | F | 4.00 | 29.40 |  | <3 | Restraint | RIA | [68] |
| *Morus serrator* | 2350 | | [56] |  |  | 33.0 | [4] |  | 7 | MF | B | F | 3.10 | 55.87 |  | <3 | Restraint | RIA | [90] |
| *Pagodroma nivea* | 419 | | [75] |  |  | 46.0 | [4] |  | 35 | MF | B | F | 4.50 | 43.15 |  |  | Restraint | RIA | [75] |
| *Parabuteo unicinctus* | 886 | | [91] |  |  |  |  |  | U | U | U | F | 8.00 | - |  |  | - | RIA | [91] |
| *Parus major* | 19 | | [68] | 21.79 | [63] | 15.4 | [4] |  | U | M | B | F | 19.90 | - |  | <3 | - | RIA | [68] |
| *Passer domesticus* | 29 | | [68] | 12.99 | [63] | 23.0 | [4] |  | U | MF | B | F | 4.50 | 33.20 |  | <3 | Restraint | RIA | [68] |
| *Passerculus sandwichensis* | 20 | | [68] | 13.90 | [4] |  |  |  | U | MF | B | F | 20.40 | 67.60 |  | <3 | Restraint | RIA | [68] |
| *Passerina cyanea* | 15 | | [68] |  |  | 11.0 | [4] |  | U | MF | B | F | 21.90 | 63.30 |  | <3 | Restraint | RIA | [68] |
| *Perdicula asiatica* | 35 | | [92] |  |  |  |  |  | 6 | M | B | F | 11.00 | 52.05 |  |  | Restraint | ELISA | [92] |
| *Petrochelidon pyrrhonota* | 23 | | [68] |  |  | 11.8 | [4] |  | U | MF | B | F | 9.00 | - |  | <3 | - | RIA | [68] |
| *Phaethon rubricauda* | 718 | | [68] | 5.61 | [86] | 32.7 | [4] |  | U | MF | B | F | 5.20 | 33.50 |  | <3 | Restraint | RIA | [68] |
| *Phalaropus fulicaria* | 56 | | [68] |  |  |  |  |  | U | MF | B | F | 21.80 | 75.40 |  | <3 | Restraint | RIA | [68] |
| *Plectrophenax nivalis* | 34 | | [68] | 13.09 | [63] | 9.6 | [4] |  | U | MF | B | F | 9.30 | 26.15 |  | <3 | Restraint | RIA | [68] |
| *Pterodroma macroptera* | 570 | | [68] |  |  |  |  |  | U | MF | B | F | 12.60 | 67.20 |  | <3 | Restraint | RIA | [68] |
| *Ptilotula penicillata* | 19 | | [68] |  |  |  |  |  | 9 | M | U | F | 15.00 | 49.48 |  | <3 | Restraint | RIA | [93] |
| *Pygoscelis adeliae* | 4850 | | [56] | 3.08 | [7] |  |  |  | 13 | MF | B | F | 7.80 | - |  | <3 | - | RIA | [94] |
| *Rissa brevirostris* | 377 | | [68] |  |  | 27.0 | [4] |  | U | M | B | F | 2.70 | 32.50 |  | <3 | Restraint | RIA | [68] |
| *Rissa tridactyla* | 411 | | [95] |  |  | 28.5 | [4] |  | U | M | B | F | 7.30 | 45.22 |  | <3 | Other | RIA | [95] |
| *Saxicola torquata* | 15 | | [68] |  |  | 8.8 | [4] |  | U | MF | B | F | 10.10 | 35.50 |  | <3 | Restraint | RIA | [68] |
| *Setophaga petechia* | 10 | | [68] |  |  | 11.0 | [4] |  | U | M | B | F | 22.30 | 86.10 |  | <3 | Restraint | RIA | [68] |
| *Setophaga ruticilla* | 8 | | [56] |  |  | 10.1 | [4] |  | 165 | MF | NB | F | 15.00 | 32.18 |  | <3 | Restraint | RIA | [96] |
| *Somateria mollissima* | 1140 | | [75] |  |  | 37.8 | [4] |  | 55 | F | B | F | 6.00 | 40.40 |  |  | Restraint | RIA | [75] |
| *Spheniscus humboldti* | 5000 | | [56] | 2.45 | [7] |  |  |  | 17 | U | B | F | 3.50 | 25.78 |  | <3 | Restraint | RIA | [76] |
| *Spheniscus magellanicus* | 4500 | | [56] |  |  | 30.0 | [4] |  | 8 | U | B | F | 4.20 | 34.68 |  | <3 | Restraint | RIA | [76] |
| *Spheniscus mendiculus* | 2500 | | [56] |  |  |  |  |  | 9 | U | B | F | 3.00 | 12.09 |  | <3 | Restraint | RIA | [76] |
| *Spinus pinus* | 17 | | [97] | 21.45 | [4] | 11.0 | [4] |  | U | MF | U | F | 8.50 | 19.68 |  | <3 | Other | RIA | [97] |
| *Spizelloides arborea* | 18 | | [68] | 19.66 | [63] | 10.8 | [4] |  | U | MF | B | F | 16.40 | 37.35 |  | <3 | Restraint | RIA | [68] |
| *Stercorarius maccormicki* | 1419 | | [75] |  |  | 23.5 | [4] |  | 31 | MF | B | F | 6.70 | - |  |  | - | RIA | [75] |
| *Sterna hirundo* | 125 | | [68] |  |  | 33.0 | [4] |  | U | MF | B | F | 10.00 | 31.60 |  | <3 | Restraint | RIA | [68] |
| *Sturnus vulgaris* | 74 | | [56] | 11.69 | [4] | 22.9 | [4] |  | U | MF | B | F | 9.90 | 33.37 |  | <3 | Restraint | EIA | [98] |
| *Sula granti* | 1754 | | [4] |  | [4] | 21.0 | [4] |  | 79 | U | NB | F | 2.10 | - |  | <3 | - | RIA | [99] |
| *Sula nebouxii* | 1542 | | [68] |  |  | 18.0 | [4] |  | U | MF | B | F | 16.10 | 37.70 |  | <3 | Restraint | RIA | [68] |
| *Sula sula* | 850 | | [100] | 4.28 | [86] | 23.0 | [4] |  | 10 | MF | NB | F | 6.20 | - |  | >3 | - | RIA | [100] |
| *Tachycineta bicolor* | 20 | | [56] |  |  | 12.1 | [4] |  | 9 | F | B | F | 8.20 | - |  | >3 | - | EIA | [101] |
| *Taeniopygia guttata* | 12 | | [68] |  |  | 12.0 | [4] |  | U | MF | B | F | 4.30 | 22.20 |  | <3 | Restraint | RIA | [68] |
| *Thalassarche melanophris* | 3552 | | [56,102] | 4.47 | [103] | 43.7 | [4] |  | 69 | MF | B | F | 2.30 | - |  | <3 | - | RIA | [102,104] |
| *Thryophilus rufalbus* | 27 | | [56] |  |  |  |  |  | 54 | MF | B | F | 9.00 | 37.83 |  | <3 | Restraint | EIA | [105] |
| *Toxostoma curvirostre* | 79 | | [56] |  |  | 10.8 | [4] |  | 26 | M | B | F | 15.00 | 39.90 |  | <3 | Restraint | RIA | [82] |
| *Turdus merula* | 113 | | [56] |  |  | 21.8 | [4] |  | 112 | MF | NB | F | 4.70 | - |  | <3 | - | EIA | [106] |
| *Uria lomvia* | 1098 | | [58] | 6.87 | [4] | 29.0 | [4] |  | 141 | U | B | F | 3.80 | 46.41 |  | <3 | Restraint | RIA | [58] |
| *Zenaida macroura* | 119 | | [56] | 5.98 | [4] | 31.3 | [4] |  | 65 | MF | B | F | 3.80 | 29.59 |  | <3 | Restraint | RIA | [107] |
| *Zonotrichia capensis* | 20 | | [68] |  |  |  |  |  | U | MF | B | F | 24.20 | 51.40 |  | <3 | Restraint | RIA | [68] |
| *Zonotrichia leucophrys* | 25 | | [68] | 12.87 | [4] | 13.3 | [4] |  | U | MF | B | F | 19.30 | 64.90 |  | <3 | Restraint | RIA | [68] |
| Mammals, cortisol dominant | |  |  |  |  |  |  |  |  |  |  |  |  |  |  |  |  |  |  |
| *Aotus trivirgatus* | 800 | | [4] | 2.73 | [4] | 30.1 | [4] |  | 7 | U | U | C | 12.00 | - | 300.00 |  | - | RIA | [108] |
| *Bos taurus* | 347000 | | [7] | 0.88 | [4] | 20.0 | [4] |  | 7 | U | U | C | 0.70 | 7.60 | 3.10 |  | ACTH | RIA | [109] |
| *Callospermophilus saturatus* | 275 | | [4] | 3.31 | [4] |  |  |  | 54 | MF | U | F | 9.60 | - | 16.80 |  | - | RIA | [110] |
| *Canis l. dingo* | 26625 | | [4] |  |  | 20.6 | [4] |  | 6 | MF | NB | C | 3.30 | 16.00 | 19.00 | <3 | ACTH | DIDA | [111] |
| *Castor fiber* | 19100 | | [112] |  |  | 20.6 | [4] |  | 34 | MF | B | F | 9.60 | - | 22.20 | >3 | - | RIA | [112] |
| *Cavia porcellus* | 728 | | [4] | 3.33 | [4] | 12.0 | [4] |  | U | M | U | C | 19.40 | - | 190.30 | <3 | - | RIA | [113] |
| *Ctenomys talarum* | 170 | | [114] | 5.05 | [4] |  |  |  | 6 | M | B | F | 2.30 | 3.13 | 20.10 | <3 | Restraint | RIA | [114] |
| *Dasyurus geoffroii* | 890 | | [4] | 2.25 | [4] | 6.2 | [4] |  | 4 | MF | NB | C | 1.50 | - | 18.00 | <3 | - | DIDA | [111] |
| *Dasyurus maculatus* | 5500 | | [4] | 1.76 | [4] | 6.8 | [4] |  | 1 | U | NB | C | 3.90 | - | 29.00 | <3 | - | DIDA | [111] |
| *Elephas maximus** | 3672000 | | [4] | 0.64 | [4] | 79.6 | [4] |  | 5 | F | B | C | 4.80 | - | 9.10 |  | - | EIA | [115] |
| *Macaca fascicularis* | 6363 | | [4] | 2.73 | [116] | 39.0 | [4] |  | 9 | U | U | C | 13.80 | - | 210.00 |  | - | RIA | [108] |
| *Macaca mulatta* | 8235 | | [4] | 2.43 | [116] | 40.0 | [4] |  | 9 | U | U | C | 8.80 | - | 245.00 |  | - | RIA | [108] |
| *Macropus giganteus* | 49500 | | [4] |  |  | 25.0 | [4] |  | 7 | MF | NB | C | 2.20 | - | 18.00 | <3 | - | DIDA | [111] |
| *Mustela p. furo* | 1140 | | [117] |  |  | 11.1 | [4] |  | 8 | MF | U | C | 2.50 | 25.80 | 26.60 |  | ACTH | RIA | [117] |
| *Notamacropus rufogriseus* | 16200 | | [4] |  |  | 15.2 | [4] |  | U | U | U | C | 1.50 | - | 17.00 | <3 | - | DIDA | [118] |
| *Odocoileus virginianus* | 87000 | | [4] | 1.99 | [4] | 23.0 | [4] |  | U | U | U | F | 2.60 | - | 56.00 |  | - | RIA | [119] |
| *Orcinus orca* | 3987500 | | [4] | 0.57 | [120] |  |  |  | 14 | M | U | C | 2.80 | - | 18.90 |  | - | EIA | [121] |
| *Oryctolagus cuniculus* | 3000 | | [122] | 3.41 | [4] | 9.0 | [4] |  | U | U | U | C | 11.00 | - | 31.50 |  | - | EIA | [122] |
| *Ovis aries* | 80000 | | [4] |  |  | 22.8 | [4] |  | U | U | NB | C | 0.90 | - | 5.20 | <3 | - | DIDA | [111] |
| *Psammomys obesus* | 212 | | [4] |  |  | 3.2 | [4] |  | U | M | U | F | 5.30 | - | 166.50 | <3 | - | RIA | [123] |
| *Saimiri sciureus* | 925 | | [4] | 5.29 | [4] | 30.2 | [4] |  | 14 | U | U | C | 20.50 | - | 1990.00 |  | - | RIA | [108] |
| *Setonix brachyurus* | 3500 | | [4] | 1.74 | [4] | 13.8 | [4] |  | 7 | MF | NB | C | 5.90 | - | 12.00 | <3 | - | DIDA | [111] |
| *Sus scrofa* | 105000 | | [124] | 0.77 | [4] | 27.0 | [4] |  | 10 | U | B | C | 11.10 | 12.05 | 17.30 |  | Other | RIA | [124] |
| *Tamias amoenus* | 48 | | [4] | 9.42 | [7] |  |  |  | 20 | F | B | F | 30.2 | 59.03 | 658.29 | <3 | Restraint | RIA | [125] |
| *Urocitellus beldingi** | 290 | | [4] | 2.71 | [4] |  |  |  | 14 | MF | U | F | 16.30 | - | 37.70 | <3 | - | RIA | [126] |
| *Urocitellus parryii* | 650 | | [7] | 4.47 | [7] |  |  |  | 27 | M | U | F | 8.10 | - | 66.20 | >3 | - | RIA | [127] |
| *Vombatus ursinus* | 25500 | | [4] |  |  | 30.0 | [4] |  | U | U | U | C | 1.30 | - | 2.30 | <3 | - | DIDA | [118] |
| *Vulpes vulpes* | 4132 | | [4] | 3.00 | [4] | 21.3 | [4] |  | U | U | NB | C | 5.90 | - | 23.00 | <3 | - | DIDA | [111] |
| *Wallabia bicolor* | 14625 | | [4] |  |  | 16.8 | [4] |  | 3 | MF | NB | C | 1.20 | - | 11.00 | <3 | - | DIDA | [111] |
| Mammals, CCST dominant |  | |  |  |  |  |  |  |  |  |  |  |  |  |  |  |  |  |  |
| *Mesocricetus auratus* | 105 | | [4] | 8.40 | [7] | 3.9 | [4] |  | U | M | U | C | 80.00 | 284.02 | 2.50 |  | Other | EIA | [128] |
| *Microtus ochrogaster* | 40 | | [4] | 8.51 | [7] | 5.3 | [4] |  | U | U | U | C | 631.00 | - |  | <3 | - | RIA | [129] |
| *Microtus pennsylvanicus* | 49 | | [4] | 10.75 | [7] |  |  |  | 77 | MF | U | F | 390.30 | - |  | <3 | - | RIA | [130] |
| *Mus musculus** | 33 | | [131] | 8.20 | [131] | 4.0 | [4] |  | 6 | F | B | C | 93.6 | 1183.00 | 12.72 |  | Restraint | RIA | [132] |
| *Tachyglossus aculeatus* | 3500 | | [4] | 0.88 | [7] | 49.5 | [4] |  | 1 | F | NB | C | 3.50 | - | 1.80 | <3 | - | DIDA | [111] |
| ^1^Stressors categorized as “Other” include synthetic angiotensin I injection, oxytocin injection, glucocorticoid implant, fasting, transport, extreme temperature, and carbon dioxide exposure. | | | | | | | | | | | | | | | | | | | |

References

1.	Herr, M.W., Graham, S.P., & Langkilde, T. (2017). Stressed snakes strike first: Hormone levels and defensive behavior in free ranging cottonmouths (Agkistrodon piscivorus). *General and Comparative Endocrinology*, *243*, 89–95. <https://doi.org/10.1016/j.ygcen.2016.11.003>

2.	Oskyrko, O., Mi, C., Meiri, S., & Du, W. (2024). ReptTraits: a comprehensive dataset of ecological traits in reptiles. *Scientific Data*, *11*. <https://doi.org/10.1038/s41597-024-03079-5>

3.	Lance, V.A., & Elsey, R.M. (1999). Plasma catecholamines and plasma corticosterone following restraint stress in juvenile alligators. *Journal of Experimental Zoology*, *283*(6), 559–565. <https://doi.org/10.1002/(sici)1097-010x(19990501)283:6><559::aid-jez7>3.0.co;2-4

4.	de Magalhaes, J.P., Abidi, Z., Dos Santos, G.A., Avelar, R.A., Barardo, D., Chatsirisupachai, K., Clark, P., De-Souza, E.A., Johnson, E.J., Lopes, I., Novoa, G., Senez, L., Talay, A., Thornton, D., & To, P.K.P. (2024). Human Ageing Genomic Resources: updates on key databases in ageing research. *Nucleic Acids Research*, *52*(D1), D900–D908. <https://doi.org/10.1093/nar/gkad927>

5.	Romero, L.M., & Wikelski, M. (2001). Corticosterone levels predict survival probabilities of Galapagos marine iguanas during El Nino events. *Proceedings of the National Academy of Sciences of the United States of America*, *98*(13), 7366–7370. <https://doi.org/10.1073/pnas.131091498>

6.	Summers, C.H., & Norman, M.F. (2005). Chronic low humidity‐stress in the lizard Anolis carolinensis: Changes in diurnal corticosterone rhythms. *Journal of Experimental Zoology*, *247*(3), 271–278. <https://doi.org/10.1002/jez.1402470311>

7.	Gillooly, J.F., Gomez, J.P., & Mavrodiev, E.V. (2017). A broad-scale comparison of aerobic activity levels in vertebrates: endotherms versus ectotherms. *Proceedings of the Royal Society B: Biological Sciences*, *284*(1849), 20162328. <https://doi.org/10.1098/rspb.2016.2328>

8.	Tokarz, R.R., McMann, S., Seitz, L., & John-Alder, H. (1998). Plasma corticosterone and testosterone levels during the annual reproductive cycle of male brown anoles (Anolis sagrei). *Physiological Zoology*, *71*(2), 139–146. <https://doi.org/10.1086/515907>

9.	Steffen, J.E., & Appel, A.G. (2012). The effect of temperature on standard metabolic rate of Brown Anoles. *Amphibia-Reptilia*, *33*(2), 297–302. <https://doi.org/10.1163/156853812x634026>

10.	Dupoué, A., Brischoux, F., Lourdais, O., & Angelier, F. (2013). Influence of temperature on the corticosterone stress-response: an experiment in the Children's python (Antaresia childreni). *General and Comparative Endocrinology*, *193*, 178–184. <https://doi.org/10.1016/j.ygcen.2013.08.004>

11.	Grassman, M., & Hess, D.L. (1992). Sex differences in adrenal function in the lizard Cnemidophorus sexlineatus: II. Responses to acute stress in the laboratory. *Journal of Experimental Zoology*, *264*(2), 183–188. <https://doi.org/10.1002/jez.1402640210>

12.	Grassman, M., & Crews, D. (1989). Ovarian and adrenal function in the parthenogenetic whiptail lizard Cnemidophorus uniparens in the field and laboratory. *General and Comparative Endocrinology*, *76*(3), 444–450. <https://doi.org/10.1016/0016-6480(89)90141-x>

13.	Dervas, E., Michalopoulou, E., Liesegang, A., Novacco, M., Schwarzenberger, F., Hetzel, U., & Kipar, A. (2023). Haematology, biochemistry and morphological features of peripheral blood cells in captive Boa constrictor. *Conservation Physiology*, *11*(1), coad001. <https://doi.org/10.1093/conphys/coad001>

14.	Barns, S.M. (2015). *Influence of food manipulation on plasma corticosterone, innate immunity, and blood chemistry in the brown African house snake (Lamprophis fuliginosus)* Middle Tennessee State University]. JEWL Digital Repository <http://jewlscholar.mtsu.edu/handle/mtsu/4756>

15.	Jackson, A.G.S., Leu, S.-Y., & Hicks, J.W. (2017). Simultaneously Occurring Elevated Metabolic States Expose Constraints in Maximal Levels of Oxygen Consumption in the Oviparous Snake Lamprophis fuliginosus. *Physiological and Biochemical Zoology*, *90*(3), 301–312. <https://doi.org/10.1086/691094>

16.	Ciocan, H., Imhof, A., Marcó, M.V.P., Isberg, S.R., Siroski, P.A., & Larriera, A. (2018). Increasing photoperiod enhances growth in captive hatchling Caiman latirostris. *Aquaculture*, *482*, 193–196. <https://doi.org/10.1016/j.aquaculture.2017.10.002>

17.	Gregory, L.F., Gross, T.S., Bolten, A.B., Bjorndal, K.A., & Guillette, L.J., Jr. (1996). Plasma corticosterone concentrations associated with acute captivity stress in wild loggerhead sea turtles (Caretta caretta). *General and Comparative Endocrinology*, *104*(3), 312–320. <https://doi.org/10.1006/gcen.1996.0176>

18.	Ultsch, G.R. (2013). Metabolic scaling in turtles. *Comparative Biochemistry and Physiology Part A: Molecular & Integrative Physiology*, *164*(4), 590–597. <https://doi.org/10.1016/j.cbpa.2013.01.012>

19.	Hişmioğullari, Ş.E., Kontaş Aşkar, T., Altuğ, M.E., & Ergün, Y. (2020). Hormonal profile of Mediterranean green turtles (Chelonia mydas). *Turkish Journal of Veterinary and Animal Sciences*, *44*(3), 588–593. <https://doi.org/10.3906/vet-1912-60>

20.	Chiari, Y., van der Meijden, A., Caccone, A., Claude, J., & Gilles, B. (2017). Self-righting potential and the evolution of shell shape in Galapagos tortoises. *Scientific Reports*, *7*(1), 15828. <https://doi.org/10.1038/s41598-017-15787-7>

21.	Schramm, B.G., Casares, M., & Lance, V.A. (1999). Steroid levels and reproductive cycle of the Galápagos Tortoise, Geochelone nigra, living under seminatural conditions on Santa Cruz Island (Galápagos). *General and Comparative Endocrinology*, *114*(1), 108–120. <https://doi.org/10.1006/gcen.1998.7240>

22.	Jessop, T.S., Tucker, A.D., Limpus, C.J., & Whittier, J.M. (2003). Interactions between ecology, demography, capture stress, and profiles of corticosterone and glucose in a free-living population of Australian freshwater crocodiles. *General and Comparative Endocrinology*, *132*(1), 161–170. <https://doi.org/10.1016/s0016-6480(03)00078-9>

23.	Gienger, C.M., Brien, M.L., Tracy, C.R., Manolis, S.C., Webb, G.J., Seymour, R.S., & Christian, K.A. (2017). Ontogenetic comparisons of standard metabolism in three species of crocodilians. *PLoS One*, *12*(2), e0171082. <https://doi.org/10.1371/journal.pone.0171082>

24.	Balment, R.J., & Loveridge, J.P. (1989). Endocrines and osmoregulatory mechanisms in the Nile crocodile, Crocodylus niloticus. *General and Comparative Endocrinology*, *73*(3), 361–367. <https://doi.org/10.1016/0016-6480(89)90192-5>

25.	Schuett, G., Taylor, E., Kirk, E., & Murdoch, W. (2004). Handling stress and plasma corticosterone levels in captive male Western Diamond-backed Rattlesnakes (Crotalus atrox). *Herpetological Review*, *35*, 229–233.

26.	Claunch, N.M., Frazier, J.A., Escallon, C., Vernasco, B.J., Moore, I.T., & Taylor, E.N. (2017). Physiological and behavioral effects of exogenous corticosterone in a free-ranging ectotherm. *General and Comparative Endocrinology*, *248*, 87–96. <https://doi.org/10.1016/j.ygcen.2017.02.008>

27.	Telemeco, R.S., & Addis, E.A. (2014). Temperature has species-specific effects on corticosterone in alligator lizards. *General and Comparative Endocrinology*, *206*, 184–192. <https://doi.org/10.1016/j.ygcen.2014.07.004>

28.	Drake, K.K., Nussear, K.E., Esque, T.C., Barber, A.M., Vittum, K.M., Medica, P.A., Tracy, C.R., Hunter, K.W., Acevedo‐Whitehouse, K., & Kristensen, T.N. (2012). Does translocation influence physiological stress in the desert tortoise? *Animal Conservation*, *15*(6), 560–570. <https://doi.org/10.1111/j.1469-1795.2012.00549.x>

29.	Moeller, K.T., Brashears, J.A., Davies, S., Demare, G., Smith, G.D., Brusch Iv, G.A., Simpson, R.K., & DeNardo, D.F. (2023). Corticosterone and immune responses to dehydration in squamate reptiles. *Journal of Experimental Biology*, *226*(23), jeb246257. <https://doi.org/10.1242/jeb.246257>

30.	Christel, C.M., DeNardo, D.F., & Secor, S.M. (2007). Metabolic and digestive response to food ingestion in a binge-feeding lizard, the Gila monster (Heloderma suspectum). *Journal of Experimental Biology*, *210*(Pt 19), 3430–3439. <https://doi.org/10.1242/jeb.004820>

31.	Moeller, K.T., Demare, G., Davies, S., & DeNardo, D.F. (2017). Dehydration enhances multiple physiological defense mechanisms in a desert lizard, Heloderma suspectum. *Journal of Experimental Biology*, *220*(Pt 12), 2166–2174. <https://doi.org/10.1242/jeb.150367>

32.	Barry, M., Cockrem, J.F., & Brunton, D.H. (2010). Seasonal variation in plasma corticosterone concentrations in wild and captive adult Duvaucel’s geckos (Hoplodactylus duvaucelii) in New Zealand. *Australian Journal of Zoology*, *58*(4), 234–242. <https://doi.org/10.1071/zo10023>

33.	Hunt, K.E., Innis, C., Merigo, C., Burgess, E.A., Norton, T., Davis, D., Kennedy, A.E., & Buck, C.L. (2019). Ameliorating transport-related stress in endangered Kemp's ridley sea turtles (Lepidochelys kempii) with a recovery period in saltwater pools. *Conservation Physiology*, *7*(1), coy065. <https://doi.org/10.1093/conphys/coy065>

34.	Jones, S.M., & Bell, K. (2004). Plasma corticosterone concentrations in males of the skink Egernia whitii during acute and chronic confinement, and over a diel period. *Comparative Biochemistry and Physiology Part A: Molecular & Integrative Physiology*, *137*(1), 105–113. <https://doi.org/10.1016/s1095-6433(03)00267-8>

35.	Natusch, D.J.D., Aust, P.W., Khadiejah, S., Ithnin, H., Isa, A., Zamzuri, C.K., Ganswindt, A., & DeNardo, D.F. (2020). Behavioral and corticosterone responses to carbon dioxide exposure in reptiles. *PLoS One*, *15*(10), e0240176. <https://doi.org/10.1371/journal.pone.0240176>

36.	Bedford, G.S., & Christian, K.A. (1999). Standard metabolic rate and preferred body temperatures in some Australian pythons. *Australian Journal of Zoology*, *46*(4), 317–328. <https://doi.org/10.1071/zo98019>

37.	Lakušić, M., Billy, G., Bjelica, V., Golubovic, A., Andelkovic, M., & Bonnet, X. (2020). Effect of capture, phenotype, and physiological status on blood glucose and plasma corticosterone levels in free-ranging dice snakes. *Physiological and Biochemical Zoology*, *93*(6), 477–487. <https://doi.org/10.1086/711958>

38.	McCallie, K.L., & Klukowski, M. (2022). Corticosterone in three species of free-ranging watersnakes: Testing for reproductive suppression and an association with body condition. *Comparative Biochemistry and Physiology Part A: Molecular & Integrative Physiology*, *269*, 111214. <https://doi.org/10.1016/j.cbpa.2022.111214>

39.	Li, C., Gu, Y., Tang, S., Fang, H., Jiang, G., & Jiang, Z. (2011). Effects of acute low temperature stress on the endocrine reactions of the Qinghai toad-headed lizard. *Current Zoology*, *57*(6), 775–780. <https://doi.org/10.1093/czoolo/57.6.775>

40.	White, C.R., Phillips, N.F., & Seymour, R.S. (2006). The scaling and temperature dependence of vertebrate metabolism. *Biology Letters*, *2*(1), 125–127. <https://doi.org/10.1098/rsbl.2005.0378>

41.	Manzo, C., Zerani, M., Gobbetti, A., Di Fiore, M.M., & Angelini, F. (1994). Is corticosterone involved in the reproductive processes of the male lizard, Podarcis sicula sicula? *Hormones and Behavior*, *28*(2), 117–129. <https://doi.org/10.1006/hbeh.1994.1009>

42.	Claunch, N.M., Bartoszek, I.A., Tillis, S., Stacy, N.I., Ossiboff, R.J., Oakey, S., Schoenle, L.A., Wellehan, J.F.X., & Romagosa, C.M. (2022). Physiological effects of capture and short-term captivity in an invasive snake species, the Burmese python (Python bivittatus) in Florida. *Comparative Biochemistry and Physiology Part A: Molecular & Integrative Physiology*, *267*, 111162. <https://doi.org/10.1016/j.cbpa.2022.111162>

43.	Kreger, M.D., & Mench, J.A. (1993). Physiological and behavioral effects of handling and restraint in the ball python (Python regius) and the blue-tongued skink (Tiliqua scincoides). *Applied Animal Behaviour Science*, *38*(3-4), 323–336. <https://doi.org/10.1016/0168-1591(93)90030-s>

44.	Dunlap, K.D., & Wingfield, J.C. (1995). External and internal influences on indices of physiological stress. I. Seasonal and population variation in adrenocortical secretion of free-living lizards, Sceloporus occidentalis. *Journal of Experimental Zoology*, *271*(1), 36–46. <https://doi.org/10.1002/jez.1402710105>

45.	Racic, A., Tylan, C., & Langkilde, T. (2020). Effects of temperature on plasma corticosterone in a native lizard. *Scientific Reports*, *10*(1), 16315. <https://doi.org/10.1038/s41598-020-73354-z>

46.	Bradshaw, S.D., Owen, F.J., & Saint Girons, H. (1988). Seasonal changes in plasma sex steroid levels in the male tuatara, Sphenodon punctatus, from Stephens Island, New Zealand. *General and Comparative Endocrinology*, *70*(3), 460–465. <https://doi.org/10.1016/0016-6480(88)90122-0>

47.	Neuman-Lee, L.A., Hudson, S.B., Webb, A.C., & French, S.S. (2020). Investigating the relationship between corticosterone and glucose in a reptile. *Journal of Experimental Biology*, *223*(Pt 2), jeb203885. <https://doi.org/10.1242/jeb.203885>

48.	Moore, I.T., & Mason, R.T. (2001). Behavioral and hormonal responses to corticosterone in the male red-sided garter snake, Thamnophis sirtalis parietalis. *Physiology & Behavior*, *72*(5), 669–674. <https://doi.org/10.1016/s0031-9384(01)00413-9>

49.	Smith, G.R. (1996). Habitat use and its effect on body size distribution in a population of the tree lizard, Urosaurus ornatus. *Journal of Herpetology*, *30*(4), 528–530. <https://doi.org/10.2307/1565696>

50.	French, S.S., Fokidis, H.B., & Moore, M.C. (2008). Variation in stress and innate immunity in the tree lizard (Urosaurus ornatus) across an urban-rural gradient. *Journal of Comparative Physiology B: Biochemical, Systemic, and Environmental Physiology*, *178*(8), 997–1005. <https://doi.org/10.1007/s00360-008-0290-8>

51.	Smith, G.D., Zani, P.A., & French, S.S. (2019). Life-history differences across latitude in common side-blotched lizards (*Uta stansburiana*). *Ecology and Evolution*, *9*(10), 5743–5751. <https://doi.org/10.1002/ece3.5157>

52.	Yao, Y.-T., Du, Y., Fang, M.-C., Lin, L.-H., & Ji, X. (2019). Developmental stage does not affect resting metabolic rate in the monitor lizard, Varanus salvator. *Animal Biology*, *69*(2), 199–212. <https://doi.org/10.1163/15707563-17000102>

53.	Jessop, T.S., Anson, J.R., Narayan, E., & Lockwood, T. (2015). An introduced competitor elevates corticosterone responses of a Native Lizard (Varanus varius). *Physiological and Biochemical Zoology*, *88*(3), 237–245. <https://doi.org/10.1086/680689>

54.	Brischoux, F., Dupoué, A., Lourdais, O., & Angelier, F. (2016). Effects of mild wintering conditions on body mass and corticosterone levels in a temperate reptile, the aspic viper (Vipera aspis). *Comparative Biochemistry and Physiology Part A: Molecular & Integrative Physiology*, *192*, 52–56. <https://doi.org/10.1016/j.cbpa.2015.11.015>

55.	Dauphin-Villemant, C., & Xavier, F. (1987). Nychthemeral variations of plasma corticosteroids in captive female Lacerta vivipara Jacquin: influence of stress and reproductive state. *General and Comparative Endocrinology*, *67*(3), 292–302. <https://doi.org/10.1016/0016-6480(87)90183-3>

56.	Dunning, J.B. (1993). *CRC Handbook of Avian Body Masses*. CRC Press.

57.	Wingfield, J.C., Deviche, P., Sharbaugh, S., Astheimer, L.B., Holberton, R., Suydam, R., & Hunt, K. (1994). Seasonal changes of the adrenocortical responses to stress in redpolls, Acanthis flammea, in Alaska. *Journal of Experimental Zoology*, *270*(4), 372–380. <https://doi.org/10.1002/jez.1402700406>

58.	Benowitz-Fredericks, Z.M., Shultz, M.T., & Kitaysky, A.S. (2008). Stress hormones suggest opposite trends of food availability for planktivorous and piscivorous seabirds in 2 years. *Deep Sea Research Part II: Topical Studies in Oceanography*, *55*(16-17), 1868–1876. <https://doi.org/10.1016/j.dsr2.2008.04.007>

59.	Deviche, P., Beouche-Helias, B., Davies, S., Gao, S., Lane, S., & Valle, S. (2014). Regulation of plasma testosterone, corticosterone, and metabolites in response to stress, reproductive stage, and social challenges in a desert male songbird. *General and Comparative Endocrinology*, *203*, 120–131. <https://doi.org/10.1016/j.ygcen.2014.01.010>

60.	Heatley, J.J., Oliver, J.W., Hosgood, G., Columbini, S., & Tully, T.N. (2000). Serum corticosterone concentrations in response to restraint, anesthesia, and skin testing in Hispaniolan Amazon parrots (Amazona ventralis). *Journal of Avian Medicine and Surgery*, *14*(3), 172–176.

61.	Harvey, S., & Phillips, J. (1980). Growth, growth hormone, and corticosterone secretion in freshwater and saline-adapted ducklings (Anas platyrhynchos). *General and Comparative Endocrinology*, *42*(3), 334–344. <https://doi.org/10.1016/0016-6480(80)90164-1>

62.	Ludders, J.W., Langenberg, J.A., Czekala, N.M., Erb, H.N., & McCormick, H. (1998). Serum corticosterone response to adrenocorticotropic hormone stimulation in Florida sandhill cranes. *Journal of Wildlife Diseases*, *34*(4), 715–721. <https://doi.org/10.7589/0090-3558-34.4.715>

63.	Lasiewski, R.C., & Dawson, W.R. (1967). A re-examination of the relation between standard metabolic rate and body weight in birds. *The Condor*, *69*(1), 13–23. <https://doi.org/10.2307/1366368>

64.	Schoech, S.J., Mumme, R.L., & Wingfield, J.C. (1997). Corticosterone, reproductive status, and body mass in a cooperative breeder, the Florida scrub-jay (Aphelocoma coerulescens). *Physiological Zoology*, *70*(1), 68–73. <https://doi.org/10.1086/639545>

65.	Quirici, V., Venegas, C.I., González-Gómez, P.L., Castaño-Villa, G.J., Wingfield, J.C., & Vásquez, R.A. (2014). Baseline corticosterone and stress response in the Thorn-tailed Rayadito (Aphrastura spinicauda) along a latitudinal gradient. *General and Comparative Endocrinology*, *198*, 39–46. <https://doi.org/10.1016/j.ygcen.2013.12.010>

66.	Romero, L.M., Holt, D.W., & Petersen, J.L. (2009). Flushing effects and seasonal changes on corticosterone levels in adult Long-Eared Owls Asio otus. *Ardea*, *97*(4), 603–608. <https://doi.org/10.5253/078.097.0428>

67.	Walker, B.G., Meddle, S.L., Romero, L.M., Landys, M.M., Reneerkens, J., & Wingfield, J.C. (2015). Breeding on the extreme edge: modulation of the adrenocortical response to acute stress in two High Arctic passerines. *Journal of Experimental Zoology Part A: Ecological Genetics and Physiology*, *323*(4), 266–275. <https://doi.org/10.1002/jez.1923>

68.	Bókony, V., Lendvai, A.Z., Liker, A., Angelier, F., Wingfield, J.C., & Chastel, O. (2009). Stress response and the value of reproduction: are birds prudent parents? *The American Naturalist*, *173*(5), 589–598. <https://doi.org/10.1086/597610>

69.	Lynn, S.E., Hunt, K.E., & Wingfield, J.C. (2003). Ecological factors affecting the adrenocortical response to stress in chestnut-collared and McCown’s longspurs (Calcarius ornatus, Calcarius mccownii). *Physiological and Biochemical Zoology*, *76*(4), 566–576. <https://doi.org/10.1086/375435>

70.	Meddle, S.L., Owen–Ashley, N.T., Richardson, M.I., & Wingfield, J.C. (2003). Modulation of the hypothalamic–pituitary–adrenal axis of an Arctic–breeding polygynandrous songbird, the Smith's longspur, Calcarius pictus. *Proceedings of the Royal Society of London B: Biological Sciences*, *270*(1526), 1849–1856. <https://doi.org/10.1098/rspb.2003.2455>

71.	Reneerkens, J., Morrison, R.G., Ramenofsky, M., Piersma, T., & Wingfield, J.C. (2002). Baseline and stress-induced levels of corticosterone during different life cycle substages in a shorebird on the high arctic breeding grounds. *Physiological and Biochemical Zoology*, *75*(2), 200–208. <https://doi.org/10.1086/340853>

72.	O'Reilly, K.M., & Wingfield, J.C. (2001). Ecological factors underlying the adrenocortical response to capture stress in arctic-breeding shorebirds. *General and Comparative Endocrinology*, *124*(1), 1–11. <https://doi.org/10.1006/gcen.2001.7676>

73.	Angelier, F., Parenteau, C., Ruault, S., & Angelier, N. (2016). Endocrine consequences of an acute stress under different thermal conditions: a study of corticosterone, prolactin, and thyroid hormones in the pigeon (Columbia livia). *Comparative Biochemistry and Physiology Part A: Molecular and Integrative Physiology*, *196*, 38–45. <https://doi.org/10.1016/j.cbpa.2016.02.010>

74.	Jung, W.J., Kim, M.S., Noh, H.J., Lee, J.W., & Yoo, J.C. (2016). Hormone profiles of obligate avian brood parasites during the breeding season. *Ibis*, *158*(2), 371–379. <https://doi.org/10.1111/ibi.12353>

75.	Tartu, S., Angelier, F., Bustnes, J.O., Moe, B., Hanssen, S.A., Herzke, D., Gabrielsen, G.W., Verboven, N., Verreault, J., & Labadie, P. (2015). Polychlorinated biphenyl exposure and corticosterone levels in seven polar seabird species. *Environmental pollution*, *197*, 173–180. <https://doi.org/10.1016/j.envpol.2014.12.007>

76.	Walker, B.G., Boersma, P.D., & Wingfield, J.C. (2014). The glucocorticoid stress response in Magellanic Penguins (Spheniscus magellanicus): comparing within and between breeding seasons, by age and colony, after fighting, and with other penguin species. *Canadian Journal of Zoology*, *93*(2), 123–131. <https://doi.org/10.1139/cjz-2014-0216>

77.	Silverin, B. (1986). Corticosterone-binding proteins and behavioral effects of high plasma levels of corticosterone during the breeding period in the pied flycatcher. *General and Comparative Endocrinology*, *64*(1), 67–74. <https://doi.org/10.1016/0016-6480(86)90029-8>

78.	Dehnhard, M., Schreer, A., Krone, O., Jewgenow, K., Krause, M., & Grossmann, R. (2003). Measurement of plasma corticosterone and fecal glucocorticoid metabolites in the chicken (Gallus domesticus), the great cormorant (Phalacrocorax carbo), and the goshawk (Accipiter gentilis). *General and Comparative Endocrinology*, *131*(3), 345–352. <https://doi.org/10.1016/S0016-6480(03)00033-9>

79.	Duckworth, R.A., Mendonça, M.T., & Hill, G.E. (2001). A condition dependent link between testosterone and disease resistance in the house finch. *Proceedings of the Royal Society of London B: Biological Sciences*, *268*(1484), 2467–2472. <https://doi.org/10.1098/rspb.2001.1827>

80.	Perfito, N., Schirato, G., Brown, M., & Wingfield, J. (2002). Response to acute stress in the harlequin duck (Histrionicus histrionicus) during the breeding season and moult: relationships to gender, condition, and life-history stage. *Canadian Journal of Zoology*, *80*(8), 1334–1343. <https://doi.org/10.1139/z02-118>

81.	Schoech, S.J., Ketterson, E.D., & Nolan, V. (1999). Exogenous testosterone and the adrenocortical response in dark-eyed juncos. *The Auk*, *116*(1), 64–72. <https://doi.org/10.2307/4089454>

82.	Fokidis, H.B., Orchinik, M., & Deviche, P. (2009). Corticosterone and corticosteroid binding globulin in birds: relation to urbanization in a desert city. *General and Comparative Endocrinology*, *160*(3), 259–270. <https://doi.org/10.1016/j.ygcen.2008.12.005>

83.	Wingfield, J.C., O'Reilly, K.M., & Astheimer, L.B. (1995). Modulation of the adrenocortical responses to acute stress in arctic birds: a possible ecological basis. *American Zoologist*, *35*(3), 285–294. <https://doi.org/10.1093/icb/35.3.285>

84.	Malueg, A., Walters, J., & Moore, I. (2009). Do stress hormones suppress helper reproduction in the cooperatively breeding red-cockaded woodpecker (Picoides borealis)? *Behavioral Ecology and Sociobiology*, *63*(5), 687–698.

85.	Landys-Ciannelli, M.M., Ramenofsky, M., Piersma, T., Jukema, J., Wingfield, J.C., & Group, C.R. (2002). Baseline and stress-induced plasma corticosterone during long-distance migration in the bar-tailed godwit, Limosa lapponica. *Physiological and Biochemical Zoology*, *75*(1), 101–110. <https://doi.org/10.1086/338285>

86.	McKechnie, A.E., & Wolf, B.O. (2004). The allometry of avian basal metabolic rate: good predictions need good data. *Physiological and Biochemical Zoology*, *77*(3), 502–521. <https://doi.org/10.1086/383511>

87.	Ellenberg, U., Setiawan, A.N., Cree, A., Houston, D.M., & Seddon, P.J. (2007). Elevated hormonal stress response and reduced reproductive output in Yellow-eyed penguins exposed to unregulated tourism. *General and Comparative Endocrinology*, *152*(1), 54–63. <https://doi.org/10.1016/j.ygcen.2007.02.022>

88.	Hennin, H.L., Wells‐Berlin, A.M., & Love, O.P. (2016). Baseline glucocorticoids are drivers of body mass gain in a diving seabird. *Ecology and Evolution*, *6*(6), 1702–1711. <https://doi.org/10.1002/ece3.1999>

89.	Wingfield, J.C. (1984). Environmental and endocrine control of reproduction in the song sparrow, Melospiza melodia: I. Temporal organization of the breeding cycle. *General and Comparative Endocrinology*, *56*(3), 406–416. <https://doi.org/10.1016/0016-6480(84)90083-2>

90.	Cockrem, J.F., Candy, E.J., Potter, M.A., & Machovsky-Capuska, G.E. (2016). Corticosterone responses to capture and restraint in Australasian Gannets, Morus serrator, at Cape Kidnappers, New Zealand. *Emu-Austral Ornithology*, *116*(1), 86–90. <https://doi.org/10.1071/MU15012>

91.	Mays, N.A., Vleck, C.M., & Dawson, J. (1991). Plasma luteinizing hormone, steroid hormones, behavioral role, and nest stage in cooperatively breeding Harris' hawks (Parabuteo unicinctus). *The Auk*, *108*(3), 619–637. <https://doi.org/10.2307/4088103>

92.	Yadav, S.K., & Haldar, C. (2014). Experimentally induced stress, oxidative load and changes in immunity in a tropical wild bird, Perdicula asiatica: involvement of melatonin and glucocorticoid receptors. *Zoology*, *117*(4), 261–268. <https://doi.org/10.1016/j.zool.2014.01.003>

93.	Buttemer, W.A., & Astheimer, L.B. (2000). Testosterone does not affect basal metabolic rate or blood parasite load in captive male white‐plumed honeyeaters Lichenostomus penicillatus. *Journal of Avian Biology*, *31*(4), 479–488. <https://doi.org/10.1034/j.1600-048X.2000.310407.x>

94.	Angelier, F., Bost, C.-A., Giraudeau, M., Bouteloup, G., Dano, S., & Chastel, O. (2008). Corticosterone and foraging behavior in a diving seabird: the Adélie penguin, Pygoscelis adeliae. *General and Comparative Endocrinology*, *156*(1), 134–144. <https://doi.org/10.1016/j.ygcen.2007.12.001>

95.	Tartu, S., Bustamante, P., Angelier, F., Lendvai, A.Z., Moe, B., Blévin, P., Bech, C., Gabrielsen, G.W., Bustnes, J.O., & Chastel, O. (2016). Mercury exposure, stress and prolactin secretion in an Arctic seabird: an experimental study. *Functional Ecology*, *30*(4), 596–604. <https://doi.org/10.1111/1365-2435.12534>

96.	Marra, P.P., & Holberton, R.L. (1998). Corticosterone levels as indicators of habitat quality: effects of habitat segregation in a migratory bird during the non-breeding season. *Oecologia*, *116*(1-2), 284–292. <https://doi.org/10.1007/s004420050590>

97.	Astheimer, L.B., Buttemer, W.A., & Wingfield, J.C. (1992). Interactions of corticosterone with feeding, activity and metabolism in passerine birds. *Ornis Scandinavica (Scandinavian Journal of Ornithology)*, *23*(3), 355–365. <https://doi.org/10.2307/3676661>

98.	Jones, B.C., Smith, A.D., Bebus, S.E., & Schoech, S.J. (2016). Two seconds is all it takes: European starlings (Sturnus vulgaris) increase levels of circulating glucocorticoids after witnessing a brief raptor attack. *Hormones and Behavior*, *78*, 72–78. <https://doi.org/10.1016/j.yhbeh.2015.10.017>

99.	Tarlow, E.M., Hau, M., Anderson, D.J., & Wikelski, M. (2003). Diel changes in plasma melatonin and corticosterone concentrations in tropical Nazca boobies (Sula granti) in relation to moon phase and age. *General and Comparative Endocrinology*, *133*(3), 297–304. <https://doi.org/10.1016/S0016-6480(03)00192-8>

100.	LormÉe, H., Jouventin, P., Trouve, C., & Chastel, O. (2003). Sex‐specific patterns in baseline corticosterone and body condition changes in breeding Red‐footed Boobies Sula sula. *Ibis*, *145*(2), 212–219. <https://doi.org/10.1046/j.1474-919X.2003.00106.x>

101.	Patterson, S.H., Winkler, D.W., & Breuner, C.W. (2011). Glucocorticoids, individual quality and reproductive investment in a passerine bird. *Animal Behaviour*, *81*(6), 1239–1247. <https://doi.org/10.1016/j.anbehav.2011.03.012>

102.	Angelier, F., Weimerskirch, H., Dano, S., & Chastel, O. (2007). Age, experience and reproductive performance in a long-lived bird: a hormonal perspective. *Behavioral Ecology and Sociobiology*, *61*(4), 611–621. <https://doi.org/10.1007/s00265-006-0290-1>

103.	Kabat, A.P., Phillips, R.A., Croxall, J.P., & Butler, P.J. (2007). Differences in metabolic costs of terrestrial mobility in two closely related species of albatross. *Journal of Experimental Biology*, *210*(16), 2851–2858. <https://doi.org/10.1242/jeb.001230>

104.	Hector, J., & Harvey, S. (1986). Corticosterone secretion through long incubation shifts in Diomedea albatrosses. *General and Comparative Endocrinology*, *62*(3), 349–352. <https://doi.org/10.1016/0016-6480(86)90043-2>

105.	Mark, M.M., & Rubenstein, D.R. (2013). Physiological costs and carry-over effects of avian interspecific brood parasitism influence reproductive tradeoffs. *Hormones and Behavior*, *63*(5), 717–722. <https://doi.org/10.1016/j.yhbeh.2013.03.008>

106.	Eikenaar, C., Müller, F., Klinner, T., & Bairlein, F. (2015). Baseline corticosterone levels are higher in migrating than sedentary common blackbirds in autumn, but not in spring. *General and Comparative Endocrinology*, *224*, 121–125. <https://doi.org/10.1016/j.ygcen.2015.07.003>

107.	Miller, D.A., Vleck, C.M., & Otis, D.L. (2009). Individual variation in baseline and stress-induced corticosterone and prolactin levels predicts parental effort by nesting mourning doves. *Hormones and Behavior*, *56*(4), 457–464. <https://doi.org/10.1016/j.yhbeh.2009.08.001>

108.	Chrousos, G., Renquist, D., Brandon, D., Eil, C., Pugeat, M., Vigersky, R., Cutler, G., Loriaux, D.L., & Lipsett, M. (1982). Glucocorticoid hormone resistance during primate evolution: receptor-mediated mechanisms. *Proceedings of the National Academy of Sciences of the United States of America*, *79*(6), 2036–2040. <https://doi.org/10.1073/pnas.79.6.2036>

109.	Gwazdauskas, F., Thatcher, W., & Wilcox, C. (1972). Adrenocorticotropin alteration of bovine peripheral plasma concentrations of cortisol, corticosterone, and progesterone. *Journal of Dairy Science*, *55*(8), 1165–1169. <https://doi.org/10.3168/jds.S0022-0302(72)85640-6>

110.	Boswell, T., Woods, S., & Kenagy, G. (1994). Seasonal changes in body mass, insulin, and glucocorticoids of free-living golden-mantled ground squirrels. *General and Comparative Endocrinology*, *96*(3), 339–346. <https://doi.org/10.1006/gcen.1994.1189>

111.	Oddie, C., Blaine, E., Bradshaw, S., Coghlan, J., Denton, D., Nelson, J., & Scoggins, B. (1976). Blood corticosteroids in Australian marsupial and placental mammals and one monotreme. *Journal of Endocrinology*, *69*(3), 341–348. <https://doi.org/10.1677/joe.0.0690341>

112.	Czerwińska, J., Chojnowska, K., Kamiński, T., Bogacka, I., Panasiewicz, G., Smolińska, N., & Kamińska, B. (2015). Plasma glucocorticoids and ACTH levels during different periods of activity in the European beaver (Castor fiber L.). *Folia Biologica*, *63*(4), 229–234. <https://doi.org/10.3409/fb63_4.229>

113.	Sachser, N., & Lick, C. (1991). Social experience, behavior, and stress in guinea pigs. *Physiology and Behavior*, *50*(1), 83–90. <https://doi.org/10.1016/0031-9384(91)90502-f>

114.	Vera, F., Antenucci, C.D., & Zenuto, R.R. (2011). Cortisol and corticosterone exhibit different seasonal variation and responses to acute stress and captivity in tuco-tucos (Ctenomys talarum). *General and Comparative Endocrinology*, *170*(3), 550–557. <https://doi.org/10.1016/j.ygcen.2010.11.012>

115.	Kajaysri, J., & Nokkaew, W. (2014). Assessment of pregnancy status of Asian elephants (Elephas maximus) by measurement of progestagen and glucocorticoid and their metabolite concentrations in serum and feces, using enzyme immunoassay (EIA). *Journal of Veterinary Medical Science*, *76*(3), 363–368. <https://doi.org/10.1292/jvms.13-0103>

116.	Leonard, W.R., Robertson, M.L., Snodgrass, J.J., & Kuzawa, C.W. (2003). Metabolic correlates of hominid brain evolution. *Comparative Biochemistry and Physiology Part A: Molecular and Integrative Physiology*, *136*(1), 5–15. <https://doi.org/10.1016/s1095-6433(03)00132-6>

117.	Rosenthal, K., Peterson, M.E., Quesenberry, K., & Lothrop, C. (1993). Evaluation of plasma cortisol and corticosterone responses to synthetic adrenocorticotropic hormone administration in ferrets. *American Journal of Veterinary Research*, *54*(1), 29–31.

118.	Coghlan, J., & Scoggins, B. (1967). Measurement of aldosterone in peripheral blood of man and sheep. *The Journal of Clinical Endocrinology and Metabolism*, *27*(10), 1470–1486. <https://doi.org/10.1210/jcem-27-10-1470>

119.	Smith, J., & Bubenik, G. (1990). Plasma concentrations of glucocorticoids in white-tailed deer: the effect of acute ACTH and dexamethasone administration. *Canadian Journal of Zoology*, *68*(10), 2123–2129. <https://doi.org/10.1139/z90-295>

120.	Worthy, G.A., Worthy, T.A., Yochem, P.K., & Dold, C. (2014). Basal metabolism of an adult male killer whale (*Orcinus orca*). *Marine Mammal Science*, *30*(3), 1229–1237. <https://doi.org/10.1111/mms.12091>

121.	O’Brien, J., Steinman, K., Fetter, G., & Robeck, T. (2017). Androgen and glucocorticoid production in the male killer whale (Orcinus orca): influence of age, maturity, and environmental factors. *Andrology*, *5*(1), 180–190. <https://doi.org/10.1111/andr.12254>

122.	Dyavolova, M., Gudev, D., Moneva, P., & Yanchev, I. (2014). Effect of high levels of ammonia in air on adrenal response to adrenocorticotropin and forced running in rabbits. *Proceedings of the International Symposium on Animal Science*, 347–353.

123.	Amirat, Z., Khammar, F., & Brudieux, R. (1980). Seasonal changes in plasma and adrenal concentrations of cortisol, corticosterone, aldosterone, and electrolytes in the adult male sand rat (Psammomys obesus). *General and Comparative Endocrinology*, *40*(1), 36–43. <https://doi.org/10.1016/0016-6480(80)90093-3>

124.	Kotwica, G., Kaminska, B., Franczak, A., Kurowicka, B., Staszkiewicz, J., Skowronski, M., Krazinski, B., & Okrasa, S. (2004). The effect of oxytocin on cortisol and corticosterone secretion in cyclic gilts-in vivo and in vitro studies. *Reproductive Biology*, *4*(1), 35–50.

125.	Kenagy, G., & Place, N.J. (2000). Seasonal changes in plasma glucocorticosteroids of free-living female yellow-pine chipmunks: effects of reproduction and capture and handling. *General and Comparative Endocrinology*, *117*(2), 189–199. <https://doi.org/10.1006/gcen.1999.7397>

126.	Mateo, J.M., & Cavigelli, S.A. (2005). A validation of extraction methods for noninvasive sampling of glucocorticoids in free-living ground squirrels. *Physiological and Biochemical Zoology*, *78*(6), 1069–1084. <https://doi.org/10.1086/432855>

127.	Boonstra, R., Hubbs, A.H., Lacey, E.A., & McColl, C.J. (2001). Seasonal changes in glucocorticoid and testosterone concentrations in free-living arctic ground squirrels from the boreal forest of the Yukon. *Canadian Journal of Zoology*, *79*(1), 49–58. <https://doi.org/10.1139/z00-175>

128.	Solomon, M.B., Sakai, R.R., Woods, S.C., & Foster, M.T. (2011). Differential effects of glucocorticoids on energy homeostasis in Syrian hamsters. *American Journal of Physiology-Endocrinology and Metabolism*, *301*(2), E307–E316. <https://doi.org/10.1152/ajpendo.00009.2011>

129.	Taymans, S.E., DeVries, A.C., DeVries, M.B., Nelson, R.J., Friedman, T.C., Castro, M., Detera-Wadleigh, S., Carter, C.S., & Chrousos, G.P. (1997). The hypothalamic–pituitary–adrenal axis of prairie voles (Microtus ochrogaster): evidence for target tissue glucocorticoid resistance. *General and Comparative Endocrinology*, *106*(1), 48–61. <https://doi.org/10.1006/gcen.1996.6849>

130.	Fletcher, Q.E., & Boonstra, R. (2006). Impact of live trapping on the stress response of the meadow vole (Microtus pennsylvanicus). *Journal of Zoology*, *270*(3), 473–478. <https://doi.org/10.1111/j.1469-7998.2006.00153.x>

131.	Speakman, J.R. (2013). Measuring energy metabolism in the mouse–theoretical, practical, and analytical considerations. *Frontiers in Physiology*, *4*, 34. <https://doi.org/10.3389/fphys.2013.00034>

132.	Gong, S., Miao, Y.-L., Jiao, G.-Z., Sun, M.-J., Li, H., Lin, J., Luo, M.-J., & Tan, J.-H. (2015). Dynamics and correlation of serum cortisol and corticosterone under different physiological or stressful conditions in mice. *PLoS One*, *10*(2), e0117503. <https://doi.org/10.1371/journal.pone.0117503>

**Figure S1:** Phylogenetic tree of all reptiles, birds, and mammals included in data analyses.

**Table S2:** Results for model selection using Bayesian Information Criterion (BIC). Model groupings are categorized by numbers 1-3 in the first column. A series of additive models were evaluated to assess the possible effects of a range of covariates on model fits. Covariates considered here included “Vert” (reptile, bird, and mammal classes), “Assay” (RIA, EIA, ELISA, or DIDA), “Time” (time to bleed at <3 min. or >3 min.), “Envr” (captive or field), “Season” (breeding or non-breeding individuals), “Sex” (males, females, or both), and “Stressor” (ACTH injections, restraint, or other). Bolded delta BIC values indicate the simplest model as the most parsimonious or having minimal difference from the most parsimonious model (∆BIC < 2.3).

|  | **df** | **BIC** | **∆BIC** |
| --- | --- | --- | --- |
| **Baseline CCST vs. Body Mass** |  |  |  |
| 1: Baseline CCST vs. Body Mass | 4 | 426.61 | **0** |
| 1: Baseline CCST vs. Body Mass + Vert | 6 | 433.45 | 6.84 |
| 2: Baseline CCST vs. Body Mass | 4 | 334.49 | **0** |
| 2: Baseline CCST vs. Body Mass + Assay | 5 | 340.11 | 5.62 |
| 2: Baseline CCST vs. Body Mass + Time | 7 | 341.53 | 7.04 |
| 3: Baseline CCST vs. Body Mass | 4 | 210.81 | **0** |
| 3: Baseline CCST vs. Body Mass + Envr | 6 | 213.70 | 2.89 |
| 3: Baseline CCST vs. Body Mass + Season | 5 | 215.74 | 4.93 |
| 3: Baseline CCST vs. Body Mass + Sex | 6 | 218.55 | 7.74 |
| **Baseline CCST vs. MSMR** |  |  |  |
| 1: Baseline CCST vs. MSMR | 4 | 242.16 | **0** |
| 1: Baseline CCST vs. MSMR + Vert | 6 | 243.06 | 0.90 |
| 2: Baseline CCST vs. MSMR | 4 | 177.30 | **0** |
| 2: Baseline CCST vs. MSMR + Assay | 7 | 179.53 | 2.23 |
| 2: Baseline CCST vs. MSMR + Time | 5 | 181.53 | 4.23 |
| 3: Baseline CCST vs. MSMR + Envr | 6 | 103.62 | 0 |
| 3: Baseline CCST vs. MSMR | 4 | 105.91 | **2.29** |
| 3: Baseline CCST vs. MSMR + Sex | 6 | 107.96 | 4.34 |
| 3: Baseline CCST vs. MSMR + Season | 5 | 108.94 | 5.32 |
| **Elevated CCST vs. Baseline CCST** |  |  |  |
| 1: Elevated CCST vs. Baseline CCST | 4 | 220.07 | **0** |
| 1: Elevated CCST vs. Baseline CCST + Vert | 6 | 228.72 | 8.65 |
| 2: Elevated CCST vs. Baseline CCST | 4 | 165.15 | **0** |
| 2: Elevated CCST vs. Baseline CCST + Time | 5 | 170.16 | 5.01 |
| 2: Elevated CCST vs. Baseline CCST + Assay | 7 | 176.68 | 11.53 |
| 3: Elevated CCST vs. Baseline CCST | 4 | 115.61 | **0** |
| 3: Elevated CCST vs. Baseline CCST + Envr | 6 | 119.24 | 3.63 |
| 3: Elevated CCST vs. Baseline CCST + Season | 5 | 121.03 | 5.42 |
| 3: Elevated CCST vs. Baseline CCST + Stressor | 6 | 122.40 | 6.79 |
| 3: Elevated CCST vs. Baseline CCST + Sex | 6 | 124.76 | 9.15 |

**Figure S2:** Plots to evaluate effects of corticosterone on lifespan for reptiles, birds, and mammals. Plots across the top row (Panels A-C) show lifespan (years) versus baseline corticosterone (CCST) concentrations (ng/ml). Plots across the bottom row (Panels D-F) show the residuals of a plot of lifespan versus mass-specific metabolic rate (MSMR; W/g) plotted against baseline corticosterone concentrations. Data are natural log-transformed, and lines are fit using ordinary least-squares (OLS) linear regression. Shading indicates 95% confidence intervals.
